# TARBP2-stablized SNHG7 regulates blood-brain barrier permeability by acting as a competing endogenous RNA to miR-17-5p/NFATC3 in Aβ-microenvironment

**DOI:** 10.1038/s41419-022-04920-8

**Published:** 2022-05-13

**Authors:** Hao Ning, Lu Zhang, Baicheng Zhu, Xinxin Zhou, Tianyuan Zhang, Teng Ma

**Affiliations:** 1 Department of Neurobiology, School of Life Sciences, China Medical University, Shenyang, 110122 China; 2 Liaoning University of Traditional Chinese Medicine, Shenyang, 110034 China

**Keywords:** Transcriptional regulatory elements, Post-translational modifications

## Abstract

Breakdown of blood-brain barrier (BBB) is recognized as serious pathological marker of Alzheimer’s disease development. Studies confirmed that β-amyloid (Aβ) deposition induced high BBB permeability by disrupting tight junction (TJ) proteins formed from endothelial cells (ECs). Here, we found TARBP2, SNHG7 and NFATC3 in expressions were increased and miR-17-5p expression was decreased in Aβ(1-42)-incubated ECs. Overexpression of TARBP2, SNHG7 and NFATC3 elevated BBB permeability and knockdown of them had converse results. Agomir-17-5p decreased BBB permeability and antagomir-17-5p increased BBB permeability. TARBP2 as a RNA-binding protein (RBP) bound to SNHG7 and resulted in longer half-life of SNHG7. The decreased expression of miR-17-5p had a negative post-transcriptional regulation to NFATC3, leading to the increased expression of NFATC3. In addition, SNHG7 regulated NFATC3 expression by acting as a molecule sponge targeting to miR-17-5p. NFATC3 inhibited TJ proteins expression by functioning as a transcription factor. TARBP2/SNHG7/miR-17-5p/NFATC3 pathway implied a potential mechanism in studies of BBB changes in AD pathological progression.

## Introduction

Blood-brain barrier (BBB), a neurovascular unit structure mainly formed by highly specialized endothelial cells membrane [1], manages the import and export homeostasis of plasma components and potential neurotoxic products from metabolism in central nervous system [2]. The breakdown of BBB integrity has been recognized as the initiating factor of different cerebral dysfunctions [3]. For Alzheimer’s disease (AD), especially, the increasing BBB permeability is determined as early marker of pathogenesis [4]. In addition, it has been widely proved that β-amyloid (Aβ) deposition, one of the major characteristics of AD development [5], induces a high BBB permeability [6–8]. In addition, though both generated from the amyloid precursor protein (APP), Aβ(1–42) is considered as the dominant form in neuronal plaques and more promising biomarker for AD comparing to Aβ(1–40) [9, 10]. Furthermore, zooming into the structure of BBB, we here focus on cerebral microvascular endothelial cells. These cells seal paracellular routes from blood and brain by forming tight junction (TJ)-related proteins [11]. And ZO-1, claudin-5 and occludin are three predominant TJ-related proteins which are significantly damaged by Aβ(1–42) judging from previous studies in Aβ(1–42)-incubated ECs [7, 11, 12].

The RNA-binding protein (RBP) is capable of significantly altering RNA fate via numerous post-transcriptional mechanisms, which has been widely regarded as a principal contributor to ECs dysfunction diseases [13]. Here, we choose trans-activation response RNA-binding protein 2 (TARBP2) as our researching target in Aβ(1–42)-incubated ECs under the reference of our sequencing work. TARBP2 as a RBP that involves in microRNA (miRNA) processing, messenger RNA (mRNA) post-transcriptional regulation [14–17]. TARBP2 is observed single-nucleotide polymorphisms that significantly associates with circulating miRNA biogenesis in AD patients [18]. What is more, TARBP2 promotes APP destabilization that relates to AD [15]. Most importantly, overexpression of TARBP2 in tumors can promote the tumor cell-induced angiogenesis [19]. Though studies mentioned above imply potential roles of TARBP2 in AD or ECs-related diseases, functions of TARBP2 on BBB dysfunction in AD microenvironment still needs to be further investigated.

A considerable amount of research has been done to explore the vital roles of long non-coding RNAs (lncRNA) in ECs-related disease and AD during the last decade [20–22]. Bioinformatic tools predict binding relationship between small nucleolar RNA host gene (SNHG7) and TARBP2. Previous studies have shown that overexpression of SNHG7 inhibits human retinal endothelial cells angiogenesis and proliferation under high glucose stimulation [23]. Moreover, in human umbilical vein endothelial cells (HUVECs) treated with oxidative low-density lipoprotein, SNHG7 is found to repress HUVECs proliferation [24]. Studies above suggest that SNHG7 is related to the property of endothelial cells. Few studies have reported roles of SNHG7 in cerebral microvascular endothelial cells in AD microenvironment. Therefore, SNHG7 is valuable to research in this study.

MiR-17-5p is identified as a key endothelial cell modulator functioning in multiple biological processes [25, 26]. For example, miR-17-5p can inhibit tube formation ability of endothelial cells in gastric cancer [27]. What’s more, miR-17-5p is confirmed that participates in BBB disruption in bacterial meningitis [27]. These studies inspirit us for the further exploration to the roles of miR-17-5p in BBB dysfunction in AD microenvironment.

Dysregulations of transcription factors (TF) in endothelial cells act as significant roles in TJ proteins expression [28–31]. We choose nuclear factor of activated T cells isoform c3 (NFATC3) via bioinformatic tool. Previous studies have demonstrated that NFATC3 functions in endothelial cells biology. For example, NFATC3 participates in endothelial dysfunction in rats exposed to chronic intermittent hypoxia [32] and diabetic mice [33]. Therefore, the roles of NFATC3 in Aβ(1–42)-incubated endothelial cells rouse our interest.

In the present study, we first detect TARBP2, SNHG7, miR-17-5p and NFATC3 endogenous expression level. Then we explore the molecular interactions mentioned above and possible mechanisms they function in to the BBB permeability changing by disrupting TJ proteins. We aim to reveal a new signaling pathway to promote AD therapy and diagnose.

## Materials and methods

### Cell cultures

Human brain microvascular endothelial cell (BMEC) line hCMEC/D3 was obtained from Dr. Couraud (Cochin Institute in Paris). Human brain normal astrocytes (NHA) were purchased from the cell bank of the Chinese Academy of Sciences in Shanghai. HCMEC/D3 cell line was maintained with EBM-2 (Lonza company, USA) culture medium for endothelial cells with fetal bovine serum, 5% fetal bovine serum (PAA Laboratories, Australia), 1% cyano-streptomycin, 1% lipid concentrate (Life Technologies, USA), 1 ng/ml bFGF, 1.4 microns of cortisol, 5 microns of ascorbic acid (Sigma-Aldrich, St. Louis, MO, USA), and 10 mM HEPES (PAA Laboratories, Australia). The medium was changed every 2 days, and the endothelial cells could grow to a monolayer in about 5–7 days and placed in an incubator at 37 °C, 5% CO_2_ and 100% humidity. HCMEC/D3 was limited from 30 to 40 passages. NHA cells were cultured in astrocyte medium RPMI-1640 (GIBCO, Carlsbad, CA, USA) and maintained at 37 °C in a humidified incubator of 5% CO_2_.

### Establishment of in vitro AD microenvironment

Human β-Amyloid(1–42)(Aβ) (GenScript, USA) was dissolved in anhydrous DSMO (2 mmol/l) on an ultraclean table and vortexed for 30 s to fully dissolve it. Next, the pre-cooled opti-MEM was added (200 μmol/l) and incubated at 4 °C for 24 h to make a stock solution. The stock solution was added to hCMEC/D3 and incubated for 48 h. The final concentration of Aβ was 5 mol/l. And other hCMEC/D3 with same density was added same stock solution without Aβ in same environment as control groups. Some cells were observed to be granular under the microscope.

### Establishment of in vitro BBB model

HCMEC/D3 was cultured in upper chamber of transwell chamber then placed in a six-well plate. Meanwhile, human glial cells were plated at a density of 2 × 10^4^ cells/ml inside the holes of the other six-well plates. When the endothelial cells were fused to 80% in the small chamber, the transwell chambers inoculated with endothelial cells were transferred to the wells of a six-well plate containing human astrocytes. These cells were called ECs (endothelial cells cocultured with astrocytes). The upper chamber was added with 1 ml of culture medium, and 2 ml of the culture medium was administered to the wells of a six-well plate. The new culture medium was replaced for 4 days. All cells were cultured in an incubator containing 5% CO_2_ and 95% air at 37 °C.

### Sample preparation for TMT-labeled proteomics analysis

Endothelial cells and Aβ(1–42)-incubated endothelial cells were added into protein lysis buffer then centrifuged at 12,000 × *g*. BCA kit was performed to measured protein concentrations and peptides were prepared from protein samples by trypsin digestion (50 μg proteins were digested by 1 μg trypsin) overnight. Subsequently, the peptides were labeled with TMT isobaric tags (Thermo Scientific, USA) at room temperature for 1 h. Samples were grouped into Abeta1 to 3 for Aβ(1–42)-incubated endothelial cells and C1 to 3 for endothelial cells. TMT-labeled proteomics data were analyzed using the Proteome Discoverer 2.2 software.

### Quantitative reverse real-time PCR (qRT-PCR)

Nanodrop Spectrophotometer (ND-100, Thermo Scientific, Waltham, MA) was used to measure the concentration and the purity of total RNA. cDNA was synthesized by reverse transcription via RT reagent Kit with gDNA Eraser (Takara, Japan), and real-time PCR was performed with TB Green Premix Ex Taq II (Takara, Japan). The primers and probes used in this study were shown in Supplementary Table S1A.

### Western blot

ECs were lysed in RIPA lysis buffer (Beyotime, Shanghai, China). The protein concentration was evaluated with BCA Protein Assay Kit (Beyotime, Shanghai, China). Protein was subjected to 10% SDS‐polyacrylamide gel electrophoresis and then transferred onto nitrocellulose membranes. Membranes were blocked with 5% nonfat milk in TBST for 2 h under room temperature and incubated overnight at 4 °C with relevant primary antibody. Antibody diluent: TARBP2 (1:500; Proteintech, USA), NFATC3 (1:1000; Proteintech, USA), ZO-1 (1:300; Thermo Scientific, Beijing, China), occludin (1:200; Thermo Scientific, Beijing, China) and claudin-5 (1:500; Thermo Scientific, Beijing, China). After three times washes, membranes were incubated in blocking buffer with a secondary antibody coupled to horseradish peroxidase for 2 h under room temperature. The complexes were detected by MicroChemi 4.2 instrument (DNR, ISRAEL) with ECL chemiluminescence kit (Beyotime, Shanghai). Results were quantified with ImageJ. The original western blot pictures were shown in Supplementary material. original western blot pictures.

### Transendothelial electric resistance (TEER) assay

Millicell-ERS (Millipore, Billerica, MA, USA) instrument was applied to detect the TEER value of in vitro BBB model according to our previous literature [34]. Electrical resistance was expressed in units of Ω cm^2^ insert using the surface area of the transwell.

### Horseradish peroxidase (HRP) flux assay

After the establishment of the in vitro BBB model, a serum-free EBM-2 medium containing 0.5 µmol/l HRP was added to the transwell chamber of the in vitro BBB model. In total, 24 h later, the culture solution in the subchamber of the BBB model was collected, HRP content was measured with an enzyme label, HRP standard curve was drawn with HRP standard, and the HRP amount infiltrated into the subchamber was calculated. HRP = the number of picomoles of HRP per square centimeter surface area per hour.

### Cell transfection

Knockdown plasmids of TARBP2, SNHG7, NFATC3 were constructed on pGPU6/GFP/Neo vector (GenePharma, Shanghai, China) and were named as TARBP2(−), SNHG7(−), NFATC3(−) groups. Overexpression plasmids of TARBP2, SNHG7, NFATC3 were constructed on pcDNA3.1 and were named as TARBP2(+), SNHG7(+), NFATC3(+) groups. Respectively, the non-targeting sequences were used as NC groups. LTX and Plus reagent (Life Technologies, Carlsbad, CA, USA) were used to stably transfect plasmids mentioned above into ECs. The primers and probes used in this study were shown in Supplementary Table S1B.

### Immunofluorescence (IF) assays

Endothelial cells were seeded at a density of 2000/cm^2^ on a 1.5% gelatin-coated cover slide. After 90% fusion, phosphate-buffered saline (PBS) was washed three times for 5 min each time and fixed with 4% paraformaldehyde for 30 min. PBS was washed three times, 5 min each time, and closed with 5% bovine serum albumin for 15 min, then incubated with corresponding antibodies overnight. PBS was used to washed cells three times for 5 min each time, then the cy3-labeled goat anti-rabbit fluorescence secondary antibody was incubated in dark for 30 min, and the nuclei were diluted and stained with DAPI at 1:500 for 10 min. PBS was used to wash cells three times every 5 min and 50% glycerol was used to seal piece. Future-proof BX60 Upright Fluorescence system was used to detect. Antibody diluent: ZO-1 (1:50; Thermo Scientific, Beijing, China), occludin (1:50; Thermo Scientific, Beijing, China) and claudin-5 (1:20; Thermo Scientific, Beijing, China).

### RNA half‐life assay

To measure the half‐life of SNHG7 transcripts, a final concentration of 5 g/ml actinomycin D (actD, MP Biomedicals) was added to the mid‐exponential cultures to inhibit transcription. At 0- 2-, 4-, 6- and 8-h post‐addition of actD, each total RNA was extracted for qRT‐PCR as described above. Taking the RNA content at 0 h post‐addition of actD as 100%, the residual RNA at each sampling time point was calculated, and the RNA half‐life (the time with 50% residual RNA) was then calibrated based on the exponential regression curve as described by Redder and Linder [35]. The primers and probes used in this study were shown in Supplementary Table S1A.

### Nascent RNA capture

Nascent RNAs were detected using Click-iT^®^ Nascent RNA Capture Kit (Thermo Fisher Scientific, USA) according to the manufacture’s protocol. Briefly, nascent RNAs were marked with 0.2 mM 5-ethymyl uridine (EU) and the EU-nascent RNA was captured on magnetic beads for subsequent qRT-PCR. The primers and probes used in this study were shown in Supplementary Table S1A.

### RNA pull-down assay

Biotin-labeled full-length SNHG7 and antisense RNA as NC were cultured with Biotin RNA Labeling Mix (GenePharma, Shanghai, China) and transfected into ECs. The RNase-free DNase I-treated biotinylated RNA was purified. RNA-protein complexes were precipitated by streptavidin-agarose beads (Invitrogen, Shanghai, China) after three times washing. The protein was detected using western blotting. GAPDH was used as control.

### Reporter vector construction and dual-luciferase reporter assays

The potential binding sequence and the corresponding mutant sequence of miR-17-5p in SNHG7, and miR-17-5p in NFATC3 3’UTR were amplified by PCR and cloned into the pmirGLO Dual-Luciferase miRNA Target Expression Vector (Promega, Madison, WI, USA) to construct wild-type and mutation-type luciferase reporter vectors (Generay Biotech Co., Shanghai, China). Human embryonic kidney cells HEK-293T were inoculated in 96-well plates. After 24 h, when the cell density was around 60–80%, the cells were co-transfected with wild-type or mutant reporter plasmids and agomir or antigomir simulators. After 48 h, luciferase activity was detected according to solution A and solution B in the luciferase detection kit (Promega, USA), and the fluorescence value of firefly was finally used as the relative luciferase activity of each group. The primers and probes used in this study were shown in Supplementary Table S1C.

### Chromatin immunoprecipitation (ChIP) assay

ECs were washed with PBS and fixed with 1% formaldehyde at room temperature for 10 min. The cross-linking reaction was quenched by adding glycine (0.1 M) and incubated for 5 min with gentle shaking. Subsequently, the cells were washed twice with cold PBS and cell lysate was prepared using ice-cold cell lysis buffer at 4 °C for 1 h. The cell lysate was sonicated for the fragmentation of chromatin to an average length of 500 to 800 bp. The samples were precleared with Protein-A agarose (Roche) by gentle rotation at 4 °C for 1 h. Then specific antibodies were added and kept at 4 °C overnight on the rotator. To capture immunoprecipitates, salmon sperm DNA (10% vol/vol) was used to block Protein-A agarose. The purified chromatin templates were amplified using qRT-PCR. The primers and probes used in this study were shown in Supplementary Table S1D.

### RNA immunoprecipitation (RIP)

The EZMagna RIP Kit (Millipore) was applied according to protocol. Complete RIP lysis buffer was used to lyse ECs. Magnetic beads conjugated with antiargonaute 2 (AGO2) or control anti-immunoglobulin G (IgG) antibody were used to incubate the cell extract. The cell extract was incubated for 6 h at 4 °C. Then, as the protein beads were removed. RT-qPCR analysis was conducted for the purification of RNA.

### Statistical analysis

All data were presented as the mean ± SD of three independent experiments. Student’s *t*-test for two groups comparisons and one-way analysis of variance with Tukey’s adjustments for multiple group comparison were performed in statistical comparison. *p* < 0.05 as a level of statistical significance. All data were analyzed by SPSS 20 software or GraphPad Prism 8.0. All data passed normality test or lognormality tests by Shapiro–Wilk test (*p* > 0.05). Parametric tests were used.

## Results

### TARBP2 was up-regulated in Aβ(1–42)-incubated ECs and increased BBB permeability

**Fig. 1:**
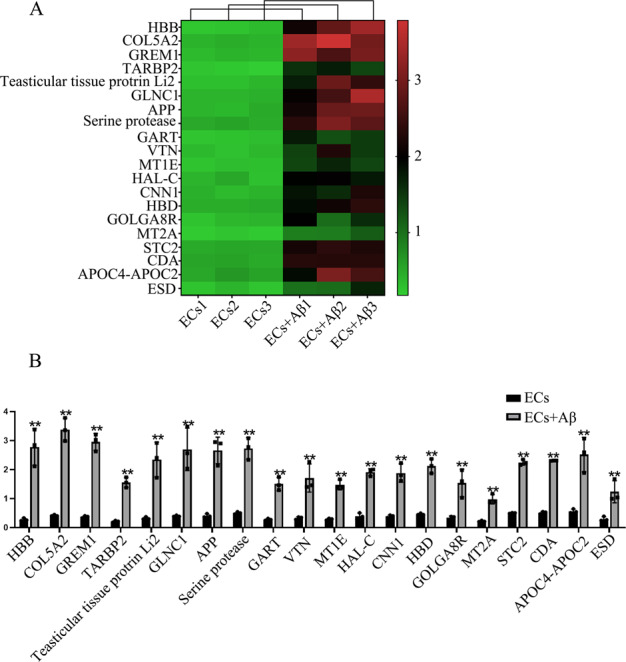
Sequencing work showed TARBP2 was up-regulated in Aβ(1–42)-incubated ECs. **A** Differential expression proteins in Aβ(1–42)-incubated ECs were visualized by heat map. **B** Quantification of differential expression proteins in Aβ(1–42)-incubated ECs compared with ECs were shown in diagram. **p < 0.01 vs. ECs group, data were presented as mean ± SD (*n* = 3, each).

**Fig. 2:**
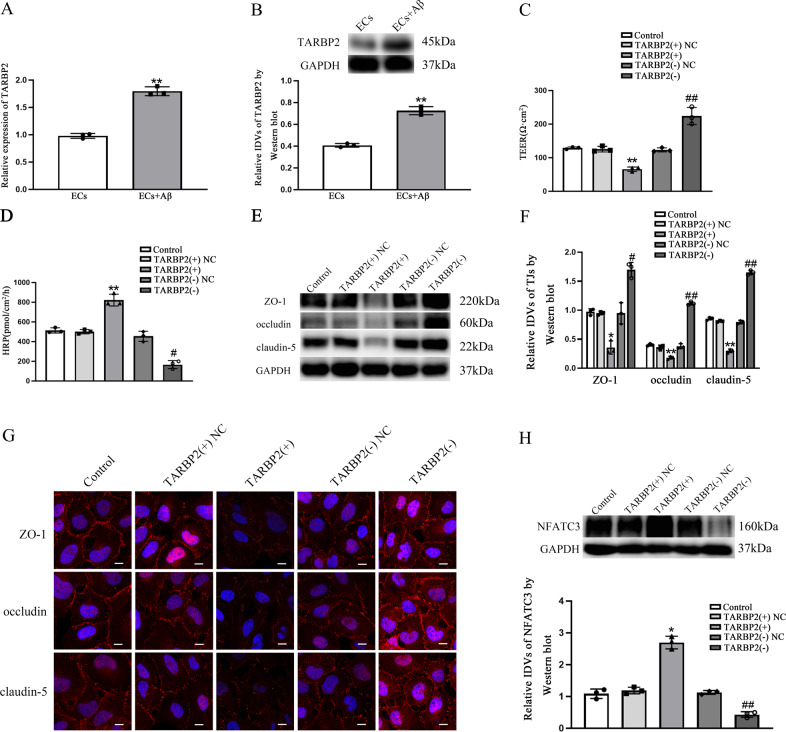
Up-regulated TARBP2 increased BBB permeability in Aβ(1–42)-incubated ECs. **A**, **B** Relative expression of TARBP2 in ECs and Aβ(1–42)-incubated ECs was detected by qRT-PCR and western blot. **p < 0.01 vs. ECs group, data were presented as mean ± SD (*n* = 3, each). **C** TEER values of BBB were presented as Ωcm^2^. **p < 0.01 vs. TARBP2(+)NC group. ^##^*p* < 0.01 vs. TARBP2(−)NC group, data were presented as mean ± SD (*n* = 3, each). **D** HRP flux was calculated as pmol/cm^2^/h. **p < 0.01 vs. TARBP2(+)NC group. ^*#*^*p* < 0.05 vs. TARBP2(−)NC group, data were presented as mean ± SD (*n* = 3, each). **E**, **F** The relative expression levels of ZO-1, occludin and claudin-5 were measured via western blot. GAPDH was used as an internal control. *p < 0.05 and **p < 0.01 vs. TARBP2(+) NC group. ^*#*^*p* < 0^.^05 and ^*##*^*p* < 0.01 vs. TARBP2(−)NC group, data were presented as mean ± SD (*n* = 3, each). **G** Immunofluorescence assay was performed to observe distribution of ZO-1, occludin and claudin-5 in ECs boundaries. ZO-1, occludin and claudin-5 were red. Nuclei were blue. Scale bar = 15 µm (*n* = 3, each). **H** The relative expression level of NFATC3 was measured via western blot. GAPDH was used as an internal control. *p < 0.05 vs. TARBP2(+) NC group. ^*##*^*p* < 0.01 vs. TARBP2(−)NC group, data were presented as mean ± SD (*n* = 3, each).

TMT-labeled quantitative proteomic analysis was performed to identified the different proteomic expression between ECs and Aβ(1–42)-incubated ECs. As shown in Fig. 1A, B, TARBP2 as a RBP in Aβ(1–42)-incubated ECs was 2.9-fold than that in ECs (*p* < 0.01). Relative expression of TARBP2 was detected by qRT-PCR and western blot. As shown in Fig. 2A, B, TARBP2 performed higher expression level in Aβ(1–42)-incubated ECs than in ECs (*p* = 0.0010 in qRT-PCR and *p* = 0.0020 in western blot). To further clarify the roles of TARBP2, we first measured BBB permeability via TEER values and HRP flux. Figure 2C, D displayed that in TARBP2(+) groups, TEER values were abated (*p* = 0.0036) and HRP flux was lifted (*p* = 0.0092) comparing with each NC group, while opposite results were obtained in TARBP(−) group vs. TARBP2(−)NC (*p* = 0.0041 in TEER values and *p* = 0.0245 in HRP flux). Next, western blot was utilized to analyze ZO-1, occludin and claudin-5 expression. ZO-1 (*p* = 0.0250), occludin (*p* = 0.0020) and claudin-5 (*p* = 0.0016) expression levels in TARBP2(+) groups were depressed vs. NC groups and promoted in TARBP2(−) comparing with NC group (*p* = 0.0112 in ZO-1, *p* = 0.0048 in occludin and *p* = 0.0090 in claudin-5) (Fig. 2E, F). Immunofluorescence assays displayed highly continuous distribution of ZO-1, occludin and claudin-5 in TARBP2(−) groups than in TARBP2(−)NC groups and reverse scenes were observed in TARBP2(+) groups vs. TARBP2(+)NC groups (Fig. 2G). Furthermore, NFATC3 was elevated in TARBP2(+) (*p* = 0.0210) comparing to NC and reduced in TARBP2(−) (*p* = 0.0018) comparing with TARBP2(−)NC groups (Fig. 2H). Based on these results, TARBP2 was confirmed having crucial roles in BBB permeability regulation.

### SNHG7 was up-regulated in Aβ(1–42)-incubated ECs and involved in BBB permeability regulation by forming binding relationship between SNHG7 and TARBP2

**Fig. 3:**
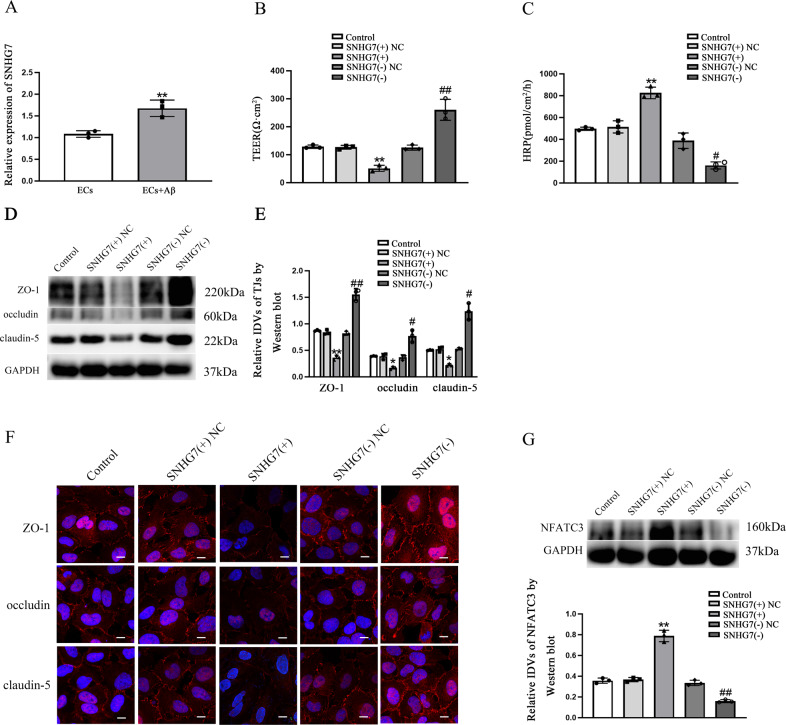
SNHG7 was induced in Aβ(1–42)-incubated ECs and increased BBB permeability. **A** Relative expression of SNHG7 in Aβ(1–42)-incubated ECs was measured by qRT-PCR. **p < 0.01 vs. ECs group, data were presented as mean ± SD (*n* = 3, each). **B** TEER values of BBB were presented as Ωcm^2^. **p < 0.01 vs. SNHG7(+)NC group. ^##^*p* < 0.01 vs. SNHG7(−)NC group, data were presented as mean ± SD (*n* = 3, each). **C** HRP flux was measured as pmol/cm^2^/h. **p < 0.01 vs. SNHG7(+)NC group. ^#^*p* < 0.05 vs. SNHG7(−)NC group, data were presented as mean ± SD (*n* = 3, each). **D**, **E** The relative expression level of ZO-1, occludin and claudin-5 was measured by western blot. GAPDH protein levels performed as an endogenous control. *p < 0.05 and **p < 0.01 vs. SNHG7(+) NC group. ^#^*p* < 0^.^05 and ^##^*p* < 0.01 vs. SNHG7(−)NC group, data were presented as mean ± SD (*n* = 3, each). **F** Immunofluorescence assay was performed to observe distribution of ZO-1, occludin and claudin-5 in ECs boundaries. ZO-1, occludin and claudin-5 were red. Nuclei were blue. Scale bar = 15 µm (*n* = 3, each). **G** The relative expression level of NFATC3 was measured via western blot. GAPDH was used as an internal control. **p < 0.01 vs. SNHG7(+) NC group. ^*##*^*p* < 0.01 vs. SNHG7(−)NC group, data were presented as mean ± SD (*n* = 3, each).

**Fig. 4:**
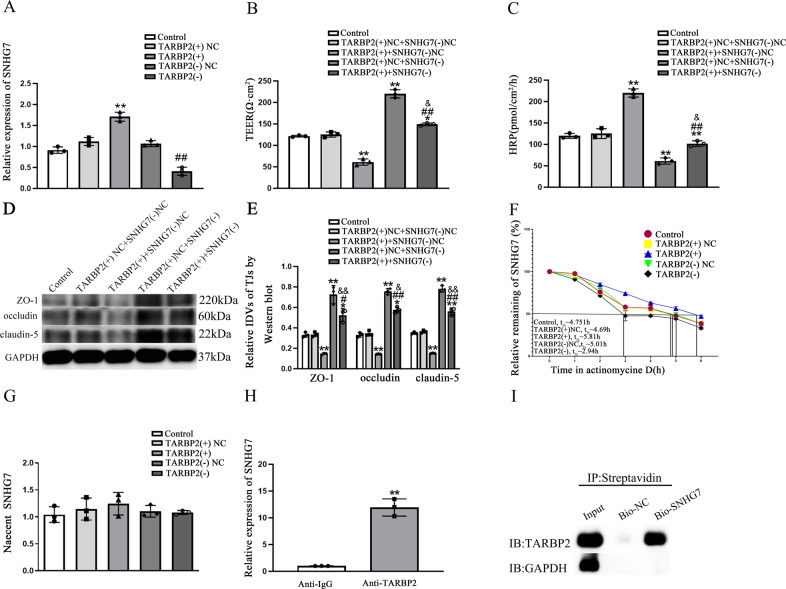
Binding relationship between SNHG7 and TARBP2 was involved in BBB permeability regulation. **A** QRT-PCR analysis of SNHG7 expression regulated by overexpression or knockdown of TARBP2 in ECs. **p < 0.01 vs. TARBP2(+)NC group. ^*##*^*p* < 0.01 vs. TARBP2(−)NC group, data were presented as mean ± SD (*n* = 3, each). **B**, **C** TEER values of BBB were presented as Ωcm^2^. HRP flux was measured as pmol/cm2/h. *p < 0.05 and **p < 0^.^01 vs. TARBP2(+)NC + SNHG7(−)NC group, ^##^*p* < 0.01 vs. TARBP2(+) + SNHG7(−)NC group, ^&^*p* < 0.05 vs. TARBP2(+)NC + SNHG7(−), data were presented as mean ± SD (*n* = 3, each). **D**, **E** The relative expression levels of ZO-1, occludin and claudin-5 were measured by western blot. GAPDH protein levels were performed as an endogenous control. *p < 0.05 and **p < 0.01 vs. TARBP2(+)NC + SNHG7(−)NC group, ^#^*p* < 0.05 and ^##^*p* < 0.01 vs. TARBP2(+) + SNHG7(−)NC group, ^&^*p* < 0.05 and ^&&^*p* < 0.01 vs. TARBP2(+)NC + SNHG7(−), data were presented as mean ± SD (*n* = 3, each). **F** Graphs showed SNHG7 levels at different times treated by ActD in TARBP2(+) group and TARBP2(−) group and respective NC group, data were presented as mean ± SD (*n* = 3, each). **G** Nascent RNA capture assays were employed to show nascent SNHG7 in TARBP2(+) group and TARBP2(−) group and respective NC group; data were presented as mean ± SD (*n* = 3, each), data were presented as mean ± SD (*n* = 3, each). **H** RNA immunoprecipitation assays (RIP) in ECs used TARBP2 antibody to enrich SNHG7. IgG was used as negative controls. **p < 0.01 vs. Anti-IgG, data were presented as mean ± SD (*n* = 3, each). **I** RNA pull-down was used to confirm SNHG7 binding to TARBP2 directly. IP was abbreviation of immunoprecipitation. IB was abbreviation of immunoblotting. GAPDH as control.

To find out lncRNA binding to TARBP2, bioinformatical websites starbase v2.0 (http://starbase.sysu.edu.cn/) [36] and RPISeq (http://pridb.gdcb.iastate.edu/RPISeq/) [37] were performed. As shown in Supplementary Fig. S1A, B, SNHG7 was chosen to further explore. With qRT-PCR, SNHG7 was discovered higher expression in Aβ(1–42)-incubated ECs vs. ECs (*p* = 0.0073) (Fig. 3A). Low TEER values (*p* = 0.0024) and high HRP flux (*p* = 0.0019) were detected in SNHG7(+) groups comparing with SNHG7(+)NC groups. On the contrary, SNHG7(−) resulted in higher TEER values (*p* = 0.0015) and lower HRP flux (*p* = 0.0240) vs. SNHG7(−)NC (Fig. 3B, C). Consistently, ZO-1 (*p* = 0.0048), occludin (*p* = 0.0480) and claudin-5 (*p* = 0.0240) expression levels were reduced in SNHG7(+) groups comparing with NC groups and induced in SNHG7(−) groups vs. SNHG7(−)NC groups (*p* = 0.0042 in ZO-1, *p* = 0.0280 in occludin and *p* = 0.0250 in claudin-5) (Fig. 3D, E). Immunofluorescence assays displayed highly continuous distribution of ZO-1, occludin and claudin-5 in SNHG7(−) groups than in SNHG7(−)NC groups and reverse scenes were observed in SNHG7(+) groups vs. SNHG7(+)NC groups (Fig. 3F). Notably, expression levels of NFATC3 in SNHG7(+) were up-regulated (*p* = 0.0020) and down-regulated in SNHG7(−) (*p* = 0.0050) comparing with each NC groups (Fig. 3G). In Fig. 4A, SNHG7 was enhanced in TARBP2(+) (*p* = 0.0020) comparing to TARBP2(+)NC and suppressed in TARBP2(−) (*p* = 0.0076) vs. TARBP2(−)NC, indicating the potential regulating relationship between TARBP2 and SNHG7. To further explore whether TARBP2 regulated BBB permeability mediated by SNHG7, the TEER values and HRP flux were performed. Firstly, in Fig. 4B, comparing with TARBP2(+)NC + SNHG7(−)NC, the TEER values were decreased in TARBP2(+) + SNHG7(−)NC (*p* = 0.0018) while increased in TARBP2(+)NC + SNHG7(−) (*p* = 0.0012) and in TARBP2(+) + SNHG7(−) (*p* = 0.0430). Furthermore, The low TEER values in TARBP2(+) + SNHG7(−)NC group were partly increased in TARBP2(+) + SNHG7(−) group (*p* = 0.0047). High TEER values in TARBP2(+)NC + SNHG7(−) group were partly decreased in TARBP2(+) + SNHG7(−) group (*p* = 0.0260). Correspondingly, comparing with TARBP2(+)NC + SNHG7(−)NC, the HRP flux was lifted in TARBP2(+) + SNHG7(−)NC (*p* = 0.0083) while diminished in TARBP2(+)NC + SNHG7(−) (*p* = 0.0046) and in TARBP2(+) + SNHG7(−) (*p* = 0.0065). Furtherly, high HRP flux in TARBP2(+) + SNHG7(−)NC group was partly reduced in TARBP2(+) + SNHG7(−) group (*p* = 0.0045). Low HRP flux in TARBP2(+)NC + SNHG7(−) group was partly lifted in TARBP2(+) + SNHG7(−) group (*p* = 0.0310) (Fig. 4C). Western blot was performed to detected ZO-1, occludin and claudin-5 expression. As shown in Fig. 4D, E, comparing to TARBP2(+)NC + SNHG7(−)NC, the expression of ZO-1 (*p* = 0.0085), occludin (*p* = 0.0036) and claudin-5 (*p* = 0.0080) in TARBP2(+) + SNHG7(−)NC was reduced while elevated in TARBP2(+)NC + SNHG7(−) (*p* = 0.0094 in ZO-1*, p* = 0.0060 in occludin and *p* = 0.0010 in claudin-5) and in TARBP2(+) + SNHG7(−) (*p* = 0.0200 in ZO-1, *p* = 0.0170 in occludin and *p* = 0.0050 in claudin-5). Furtherly, low TJ-related proteins expressions in TARBP2(+) + SNHG7(−)NC group were partly enhanced in TARBP2(+) + SNHG7(−) group (*p* = 0.0150 in ZO-1, *p* = 0.0020 in occludin and *p* = 0.0022 in claudin-5). High TJ-related proteins expressions in TARBP2(+)NC + SNHG7(−) group were partly attenuated in TARBP2(+) + SNHG7(−) group (*p* = 0.0080 in ZO-1, *p* = 0.0340 in occludin and *p* = 0.0094 in claudin-5). Moreover, as shown in Fig. 4F, the half-life of SNHG7 was significantly up-regualted in TARBP2(+) group and suppressed in TARBP2(−) group comparing to each NC groups. However, nascent SNHG7 level in each group was not statistically significant (*p* = 0.5870 in TARBP2(+), *p* = 0.7410 in TARBP2(−)) (Fig. 4G). To explore whether TARBP2 could directly bind to SNHG7, RIP and RNA pull-down assays were conducted. As shown in Fig. 4H, SNHG7 enrichment in anti-TARBP2 was 11.9-fold than that in anti-IgG (*p* = 0.0030) and TARBP2 was precipitated by bio-SNHG7 in Fig. 4I, demonstrated a directly binding relationship between TARBP2 and SNHG7.

### MiR-17-5p was down-regulated in Aβ(1–42)-incubated ECs, and down-regulated miR-17-5p by TARBP2 with the mediation of SNHG7 regulation increased BBB permeability

**Fig. 5:**
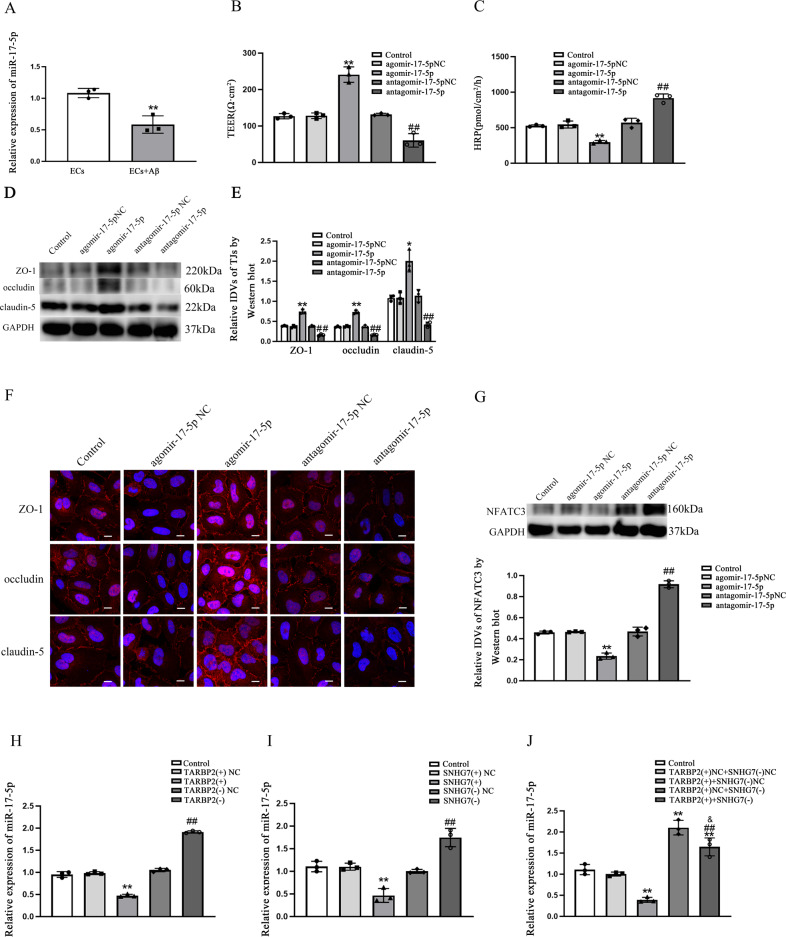
MiR-17-5p was down-regulated in Aβ(1–42)-incubated ECs, and down-regulated miR-17-5p by TARBP2 with the mediation of SNHG7 regulation increased BBB permeability. **A** Relative expression of miR-17-5p in ECs and Aβ(1–42)-incubated ECs was detected by qRT-PCR and western blot. **p < 0.01 vs. ECs group, data were presented as mean ± SD (*n* = 3, each). **B** TEER values of BBB were presented as Ωcm^2^. **p < 0.01 vs. agomiR-17-5p NC group. ^##^*p* < 0.01 vs. antagomiR-17-5p NC group. **C** HRP flux was calculated as pmol/cm^2^/h. **p < 0.01 vs. agomiR-17-5p NC group. ^*##*^*p* < 0.01 vs. antagomiR-17-5p NC group, data were presented as mean ± SD (*n* = 3, each). **D**, **E** The relative expression levels of ZO-1, occludin and claudin-5 were measured by western blot. GAPDH was used as an internal control. *p < 0.05 and **p < 0.01 vs. agomiR-17-5p NC group. ^*##*^*p* < 0.01 vs. antagomiR-17-5p NC group, data were presented as mean ± SD (*n* *=* 3, each). **F** Immunofluorescence assay was performed to observe distribution of ZO-1, occludin and claudin-5 in ECs boundaries. ZO-1, occludin and claudin-5 were red. Nuclei were blue. Scale bar = 15 µm (*n* = 3, each). **G** The relative expression level of NFATC3 was measured via western blot. GAPDH was used as an internal control. **p < 0.01 vs. agomiR-17-5p NC group. ^##^*p* < 0.01 vs. angomiR-17-5p NC group, data were presented as mean ± SD (*n* = 3, each). **H** miR-17-5p expression was regulated by overexpression or knockdown of TARBP2 in ECs with qRT-PCR analysis. **p < 0.01 vs. TARBP2(+)NC group. ^##^*p* < 0.01 vs. TARBP2(−)NC group, data were presented as mean ± SD (*n* = 3, each). **I** miR-17-5p expression was regulated by overexpression or knockdown of SNHG7 in ECs with qRT-PCR analysis. **p < 0.01 vs. SNHG7(+)NC group. ^##^*p* < 0.01 vs. SNHG7(−)NC group, data were presented as mean ± SD (*n* = 3, each). **J** miR-17-5p expression was regulated by TARBP2 with mediation of SNHG7 in qRT-PCR analysis. **p < 0.01 vs. TARBP2(+)NC + SNHG7(−)NC group, ^##^*p* < 0.01 vs. TARBP2(+) + SNHG7(−)NC group, ^&^*p* < 0.05 vs. TARBP2(+)NC + SNHG7(−), data were presented as mean ± SD (*n* = 3, each).

The prediction of miRNAs targeting to SNHG7 was performed by DIANA database (http://carolina.imis.athena-innovation.gr/diana_tools) [38] (Supplementary Fig. S1C). And miR-17-5p was significant down-regulated in Aβ(1–42)-incubated ECs comparing with ECs (*p* = 0.0053) (Fig. 5A). To investigate the function of miR-17-5p in ECs, agomiR-17-5p and antagomiR-17-5p were transiently transfected into ECs. Figure 5B, C illustrated that TEER values were elevated and HRP flux was reduced in agomiR-17-5p groups (*p* = 0.0088 in TEER values and *p* = 0.0065 in HRP flux) vs. NC. However, lower TEER values and higher HRP flux were detected in antagomiR-17-5p groups (*p* = 0.0067 in TEER values and *p* = 0.0045 in HRP flux) vs. NC group. Western blot presented that ZO-1, occludin and claudin-5 were promoted in agomiR-17-5p groups (*p* = 0.0027 in ZO-1, *p* = 0.0022 in occludin and *p* = 0.0110 in claudin-5) and diminished in antagomiR-17-5p groups (*p* = 0.0039 in ZO-1, *p* = 0.0080 in occludin and *p* = 0.0080 in claudin-5) comparing to each NC groups (Fig. 5D, E). Immunofluorescence assays were observed high continuous contribution in agomiR-17-5p groups and discontinuous contribution in antagomiR-17-5p groups comparing with NC groups (Fig. 5F). NFATC3 expression was inhibited in agomiR-17-5p (*p* = 0.0020) and activated in antagomiR-17-5p (*p* = 0.0010) vs. each NC groups (Fig. 5G).

In addition, qRT-PCR results presented that the expression of miR-17-5p was inhibited in TARBP2(+) (*p* = 0.0021) and SNHG7(+) (*p* = 0.0024) but prompted in TARBP2(−) (*p* = 0.0015) and SNHG7(−) (*p* = 0.0036) in compared with each NCs (Fig. 5H, I). Furthermore, as shown in Fig. 5J, comparing with TARBP2(+)NC + SNHG7(−)NC, miR-17-5p in TARBP2(+) + SNHG7(−)NC was diminished (*p* = 0.0032) while raised in TARBP2(+)NC + SNHG7(−) (*p* = 0.0057) and in TARBP2(+) + SNHG7(−) (*p* = 0.0010). Next, the down-regulated miR-17-5p in TARBP2(+) + SNHG7(−)NC group was partly lifted in TARBP2(+) + SNHG7(−) group (*p* = 0.0060). Up-regulated miR-17-5p in TARBP2(+)NC + SNHG7(−) group was partly repressed in TARBP2(+) + SNHG7(−) group (*p* = 0.026), showing that the TARBP2 impaired miR-17-5p expression by SNHG7 mediating.

### NFATC3 was up-regulated in Aβ(1–42)-incubated ECs to facilitated the increase of BBB permeability, and NFATC3 was direct target of miR-17-5p

**Fig. 6:**
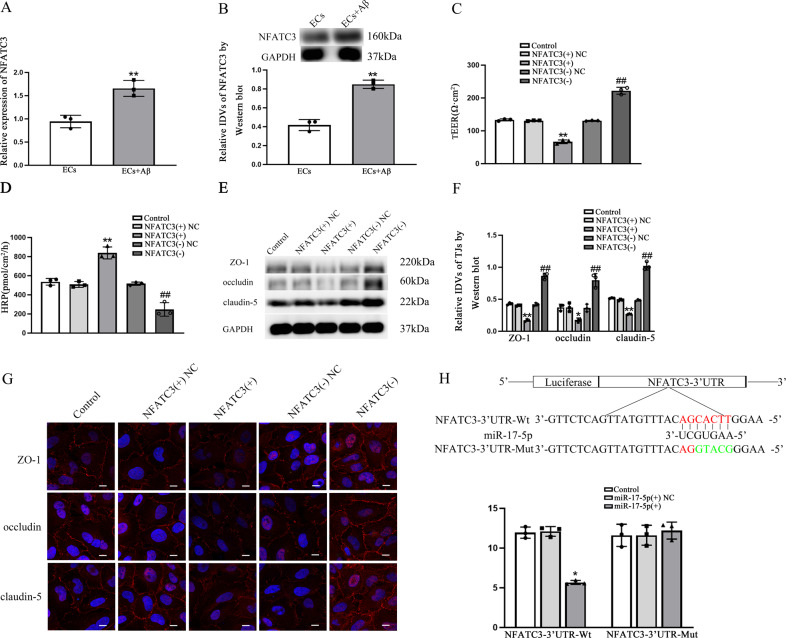
NFATC3 was up-regulated in Aβ(1–42)-incubated ECs to facilitated the increase of BBB permeability, and NFATC3 was direct target of miR-17-5p. **A**, **B** Relative expression of NFATC3 in ECs and Aβ(1–42)-incubated ECs was detected by qRT-PCR and western blot. **p < 0.01 vs. ECs group, data were presented as mean ± SD (*n* = 3, each). **C** TEER values of BBB were presented as Ωcm^2^. **p < 0.01 vs. NFATC3(+)NC group. ^##^*p* < 0.01 vs. NFATC3(−)NC group, data were presented as mean ± SD (*n* = 3, each). **D** HRP flux was calculated as pmol/cm^2^/h. **p < 0.01 vs. NFATC3(+)NC group. ^*#*#^*p* < 0.01 vs. NFATC3(−)NC group, data were presented as mean ± SD (*n* = 3, each). **E**, **F** The relative expression levels of ZO-1, occludin and claudin-5 were measured via western blot. GAPDH was used as an internal control. *p < 0.05 and **p < 0.01 vs. NFATC3(+)NC group. ^##^*p* < 0^.^01 vs. NFATC3(−)NC group^,^ data were presented as mean ± SD (*n* = 3, each). **G** Immunofluorescence assay was performed to observe distribution of ZO-1, occludin and claudin-5 in ECs boundaries. ZO-1, occludin and claudin-5 were red. Nuclei were blue, Scale bar = 15 µm (*n* = 3, each). **H** Dual-luciferase reporter assays were utilized to measure the binding relationship between miR-17-5p and 3’UTR of NFATC3. *p < 0.05 vs. miR-17-5p(+) NC, data were presented as mean ± SD (*n* = 3, each).

DIANA database was utilized to predict miRNAs targeting to NFATC3 (Supplementary Fig. S1D) and results showed that NFATC3 was direct target of miR-17-5p with the specific binding site being located at the seed sequence. QRT-PCR and western blot were performed to analyzed NFATC3 expression level. As shown in Fig. 6A, B, NFATC3 was up-regulated in Aβ(1–42)-incubated ECs vs. ECs (*p* = 0.0039 in qRT-PCR and *p* = 0.0050 in western blot). In NFATC3(+) group, lower TEER values (*p* = 0.0059) and higher HRP flux (*p* = 0.0094) were observed comparing with each NC. Up-regulated TEER values (*p* = 0.0048) and down-regulated HRP flux (*p* = 0.0047) were presented in NFATC3(−) group vs. each NC group (Fig. 6C, D). ZO-1, occludin and claudin-5 expressions were inhibited in NFATC3(+) (*p* = 0.0040 in ZO-1, *p* = 0.0140 in occludin and *p* = 0.0026 in claudin-5) and activated in NFATC3(−) (*p* = 0.0020 in ZO-1, *p* = 0.0052 in occludin and *p* = 0.0034 in claudin-5) comparing with NC groups (Fig. 6E, F). Immunofluorescence assays showed highly continuous distribution of ZO-1, occludin and claudin-5 in NFATC3(−) groups than that of NFATC3(−)NC groups and reverse scenes were observed in NFATC3(+) groups vs. NFATC3(+)NC groups (Fig. 6G). Luciferase activities in NFATC3-3’UTR-Wt were inhibited (*p* = 0.0200) comparing with miR-17-5p(+)NC but there were no significant differences of luciferase activities in NFATC3-3’UTR-Mut (*p* = 0.9760) comparing with miR-17-5p(+)NC (Fig. 6H).

### SNHG7 acting as a miR-17-5p ceRNA to regulate NFATC3 expression

**Fig. 7:**
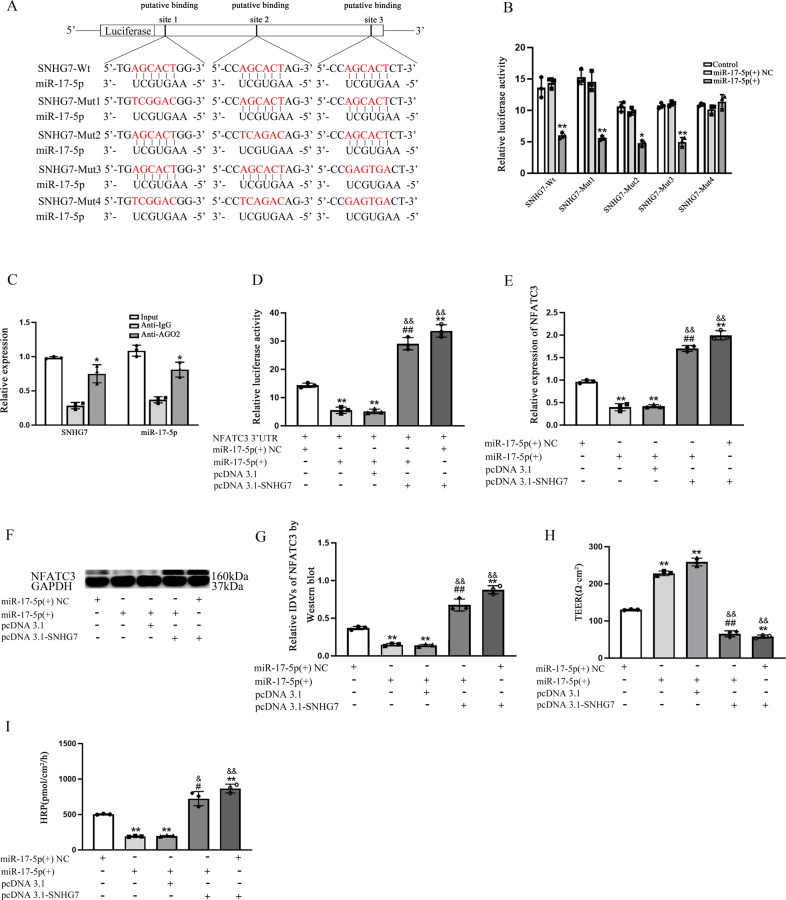
SNHG7 acting as a miR-17-5p ceRNA to regulate NFATC3 expression. **A**, **B** Dual-luciferase reporter assays were performed to detect putative binding sites between miR-17-5p and SNHG7. *p < 0.05 and **p < 0.01 vs. miR-17-5p(+)NC, data were presented as mean ± SD (*n* = 3, each). **C** RIP assays were applied to identify miR-17-5p in SNHG7-RISC complex. *p < 0.05 vs. anti-IgG group, data were presented as mean ± SD (*n* = 3, each). **D** Dual-luciferase reporter assays were utilized to measure the competing endogenous RNA activity of SNHG7. **p < 0.01 vs. miR-17-5p(+) NC group. ^##^*p* < 0.01 vs. miR-17-5p(+) + pcDNA3.1 groups^. &&^*p* < 0.01 vs. miR-17-5p(+) group, data were presented as mean ± SD (*n* = 3, each). **E**–**G** The relative expression level of NFATC3 was measured via qRT-PCR and western blot. GAPDH was used as an internal control. **p < 0.01 vs. miR-17-5p(+)NC group. ^##^*p* < 0.01 vs. miR-17-5p(+) + pcDNA3.1 groups. ^&&^*p* < 0.01 vs. miR-17-5p(+) group, data were presented as mean ± SD (*n* = 3, each). **H**, **I** TEER values of BBB were presented as Ωcm^2^. HRP flux was calculated as pmol/cm^2^/h. **p < 0.01 vs. miR-17-5p(+) NC group. ^#^*p* < 0.05 and ^##^*p* < 0.01 vs. miR-17-5p(+) + pcDNA3.1 groups. ^&^*p* *<* 0.05 and ^&&^*p* < 0.01 vs. miR-17-5p(+) group, data were presented as mean ± SD (*n* = 3, each).

Three miRNA-binding sites were predicted in SNHG7 sequence, which might indicate the molecule sponge role of SNHG7 to miR-17-5p. Results of dual-luciferase reporter assay further corroborated our expectation. Lower luciferase activities were detected in SNHG7-Wt (*p* = 0.0016), SNHG7-Mut1 (*p* = 0.0069), SNHG7-Mut2 (*p* = 0.0130) and SNHG7-Mut3 (*p* = 0.0065) comparing with each NC and there were no significant differences in SNHG7-Mut4 (*p* = 0.8150) (Fig. 7A, B). With RIP assay, the enrichment of SNHG7 and miR-17-5p in anti-AGO was significantly higher than that of in anti-IgG (*p* = 0.0370 in SNHG7 and *p* = 0.0210 in miR-17-5p) (Fig. 7C), which indicated that miR-17-5p and SNHG7 could form miRNA ribonucleoprotein complexes (miRNPs) with AGO protein. To further reveal the ceRNA mechanism regulating NFATC3 expression, NFATC3 3’UTR was inserted and transfected into HEK-293T cells accompanied by miR-17-5p or SNHG7 vector. As shown in Fig. 7D, comparing to miR-17-5p(+)NC group, the luciferase activities of NFATC3 3’UTR were restrained in miR-17-5p(+) (*p* = 0.0027) and miR-17-5p(+) + pcDNA3.1 (*p* = 0.0056) groups. Notably, comparing to miR-17-5p(+) + pcDNA3.1, miR-17-5p(+) + SNHG7 partly rescued the low luciferase activities (*p* = 0.0020). The expression of NFATC3 mRNA and protein was significantly inhibited in miR-17-5p(+) group (*p* = 0.0024 in qRT-PCR, *p* = 0.0016 in western blot) and pcDNA3.1 + miR-17-5p(+)group (*p* = 0.0014 in qRT-PCR and *p* = 0.0076 in western blot), then SNHG7 partly rescued this tendency in miR-17-5p(+) + SNHG7 (*p* = 0.0067 in qRT-PCR and *p* = 0.0018 in western blot) comparing with miR-17-5p(+) + pcDNA3.1 (Fig. 7E–G). TEER values and HRP flux were used to measure BBB permeability. As shown in Fig. 7H, I, up-regulated TEER values and down-regulated HRP flux were detected in miR-17-5p(+) group (*p* = 0.0067 in TEER values and *p* = 0.0081 in HRP flux) and miR-17-5p(+) + pcDNA3.1 group (*p* = 0.0015 in TEER values and *p* = 0.0031 in HRP flux). SNHG7 partly rescued these tendency in miR-17-5p(+) + SNHG7 (*p* = 0.0070 in TEER values and *p* = 0.0150 in HRP flux) vs. miR-17-5p(+) + pcDNA3.1.

### NFATC3 inhibited TJ-related proteins expression by acting as a transcription factor

**Fig. 8:**
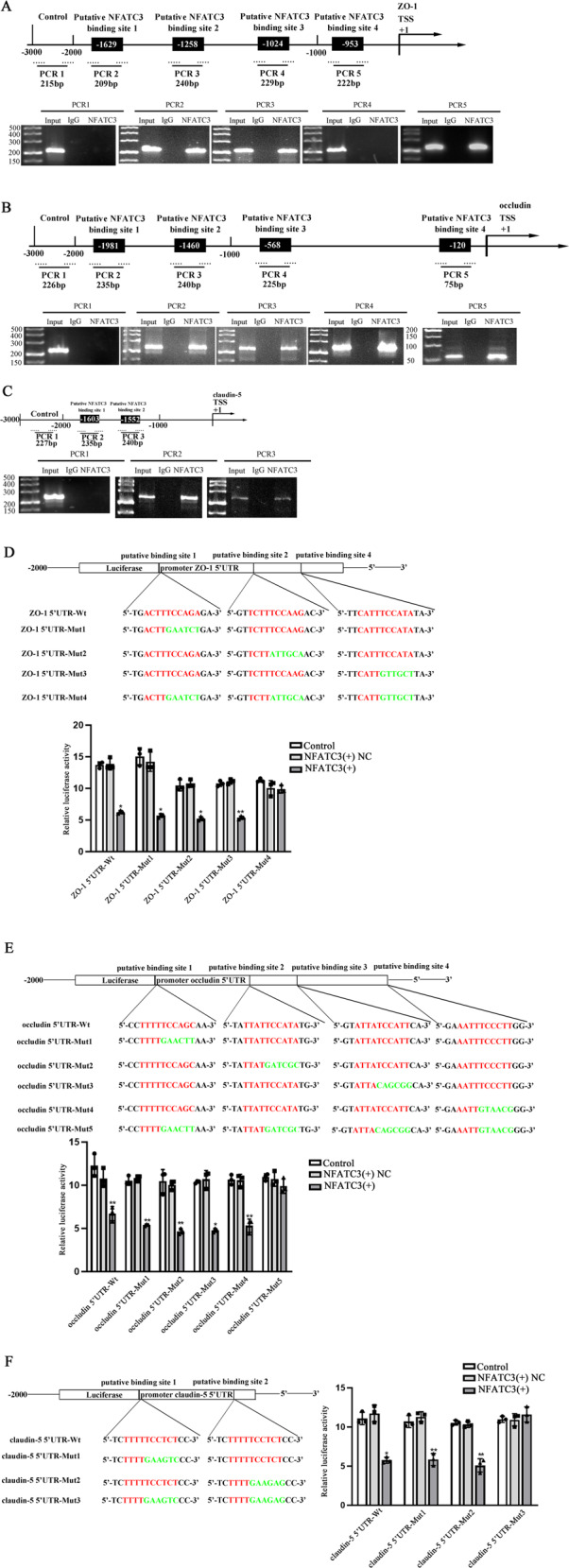
NFATC3 inhibited TJ-related proteins expression by acting as a transcription factor. **A**–**C** Diagram above represented the predicted promoter regions of ZO-1, occludin and claudin-5 in 3000 bp upstream of the transcription start site (TSS, designated as +1). Chromatin immunoprecipitation (ChIP) PCR was constructed for the binding sites and upstream regions not expected to interact with NFATC3 were used as negative control. Images below showed the PCR results by agarose gel electrophoresis. **D**–**F** Dual-luciferase reporter assays were applied to test the binding sites of NFATC3 with ZO-1, occludin and claudin-5. *p < 0.05 and **p < 0.01 vs. NFATC3(+)NC group, data were presented as mean ± SD (*n* = 3, each).

Four binding sites in ZO-1 promoter, four binding sites in occludin promoter and two binding sites in claudin-5 promoter of NFATC3 were predicted by JASPAR (Supplementary Fig. S1E–G). ChIP assays were applied to test these putative binding sites. As shown in Fig. 8A–C, NFATC3 was recruited by binding sites 1,2,4 in ZO-1 promoter, binding sites 1,2,3,4 in occludin promoter and binding sites 1,2 in claudin-5 promoter.

To further confirm these sites, dual-luciferase reporter assays were performed. As shown in Fig. 8D–F lower luciferase activities in ZO-1 5’UTR-Wt (*p* = 0.0360) and ZO-1 5’UTR-Mut1 (*p* = 0.0310), 2 (*p* = 0.0150) and 3 (*p* = 0.0060) were detected comparing to each NC. But there were no significant differences between ZO-1 5’UTR-Mut4 (*p* = 0.1270) and NC. Similarly, luciferase activities in occludin 5’UTR-Wt (*p* = 0.0058) and occludin 5’UTR-Mut1 (*p* = 0.0044), 2 (*p* = 0.0035), 3 (*p* = 0.0168) and 4 (*p* = 0.0031) were inhibited comparing to each NC. But there were no significant differences between occludin 5’UTR-Mut5 (*p* = 0.2670) and NC. Furthermore, claudin-5 5’UTR-Wt (*p* = 0.0200) and claudin-5 NFATC3 5’UTR-Mut1 (*p* = 0.0020), 2 (*p* = 0.0048) presented lower luciferase activities vs. each NC and there were no significant differences between claudin-5 5’UTR-Mut3 (*p* = 0.3210) and NC. Results above demonstrated that NFATC3 could bind to promoters of TJ-related proteins to suppress their expression.

Finally, As shown in Supplementary Fig. S2, TARBP2 stabilizes SNHG7/miR-17-5p/NFATC3 signaling pathway to regulate BBB permeability in Aβ-microenvironment.

## Discussion

It brings little effects that numerous clinical trials have been paid to explore methods against Aβ generation or facilitate Aβ clearance [39]. Therefore, treatment for BBB leakage, as an incipient marker of AD, is attracting considerable attention [1]. In the present study, we first identified that TARBP2 was up-regulated in Aβ(1–42)-incubated ECs, and the increased expression of TARBP2 enhanced the permeability of BBB. We provided further evidence for that TARBP2 could stabilize SNHG7 by binding to SNHG7, which resulted in the up-regulation of SNHG7 in Aβ(1–42)-incubated ECs. Hence, Up-regulated SNHG7 as a ceRNA bound to miR-17-5p for competing with NFATC3, thereby further inhibiting the function of miR-17-5p and promoting the NFATC3 exression. Furthermore, the increased NFATC3 restrained TJ-related proteins expression by acting as a TF, thus modulating the permeability of BBB. To the best of our knowledge, this was the first report about the study on regulatory mechanism of BBB permeability in AD using signaling pathway consisted of TARBP2, SNHG7, miR-17-5p and NFATC3 (Supplementary Fig. S2).

RBPs have been recognized as a crucial player in AD development [40]. For example, overexpression of RBP-HNRNPU bound to BACE1 mRNA to regulate the stability of BACE1 mRNA hence resulted in learning and memory abilities impairment in AD rat [41]. Our study revealed that RBP-TARBP2 significantly elevated in Aβ(1–42)-incubated ECs and participated in BBB permeability regulation. TARBP2 increased SNHG7 and NFATC3 expression then further disrupted BBB integrity in ECs. In parallel with our results, other studies showed that RBP-hnRNPK could interact with lncRNA-PUNISHER to modulate PUNISHER loading to extracellular vesicle thus regulating ECs migration and proliferation [42].

LncRNA modulates BBB permeability by regulating TJ-related proteins [43, 44]. We first identified high expression of SNHG7 in Aβ(1–42)-incubated ECs impaired the integrity of BBB. Similarly, up-regulated lnc00094 in Aβ-incubated ECs impaired TJ-related proteins expression by inhibited Endophilin-1 expression [45]. Inconsistent with our finding, highly expressed lncRNA-TUG1 decreased BTB permeability by promoting TJ-related proteins expression. Based on these results, lncRNA was closely related to the structure and function of endothelial cells in BBB and BTB.

The abnormal alterations of RBP expression and activity relate to lncRNA post-transcriptional remodeling and RNA stability. The interactions between RBP and RNA played significant roles in ECs [13, 46]. In the present study, TARBP2 was confirmed to bind to SNHG7 directly and stabilized SNHG7 via increasing the half-life of it. Consistent with our results, RBP-TRA2A bound to linc00662 and prolonged the half-life of linc00662 in AD microenvironment to regulate BBB permeability [47]. The above research showed that the interactions between RBP and RNA played significant roles in ECs of BBB and BTB.

miRNA has been the subject of many classic studies in AD and in ECs-related diseases [43, 48, 49]. In the present study, we elucidated that miR-17-5p was decreased in Aβ(1–42)-incubated ECs that increased BBB permeability. Consistently, lessened miR-92b in oxygen-glucose deprivation-induced BMECs disrupted BBB integrity [50]. Conversely, low expression of miR-153 and miR-377 in glioma-exposed endothelial cells (GECs) targeted to TF-FOXR2 to induce TJ-related proteins expression and enhance BBB integrity [51]. The reason for the different results of the above experiment might be that different miRNAs in different tissues had tissue specificity and exerted different physiological functions.

TF can bind to specific promoter regions of TJ-related proteins to regulate TJ-related proteins expression [47, 52]. NFATC3 acting as a TF has been researched widely in EC biology. Our team members found TF-NFATC3 could suppress TJ-related proteins expression by which regulated BBB permeability. Similar to our results, in diabetic mice, elevated NFATC3 led to microvascular endothelial cells dysfunction [33]. Interactions between miRNA and TFs were reported widely in ECs-related diseases [43]. We revealed that miR-17-5p could bind to 3’UTR of NFATC3 mRNA and blocked NFATC3 expression in ECs to prompt TJ-related proteins expression. Inconsistent with our finding, miR-18a bound to 3’UTR of TF-RUNX1 mRNA to inhibit RUNX1 expression but ZO-1 and occludin were therefore decreased [53]. These results implied that TFs and miRNA/TFs interactions played multifarious roles in BBB permeability regulation.

CeRNA crosstalks are involved into interactions between ncRNA and proteins, which have been widely observed in ECs-related diseases [54]. Here, we found SNHG7 as a miR-17-5p ceRNA could regulate NFATC3 expression. Consistent with our results, in GECs, lncRNA-MIAT functioned as a miR-140-3p ceRNA to induce ZAK expression, thereby prompting BTB permeability [34]. In addition, increased LINC00094 in Aβ(1–42)-incubated ECs sponged miR-224-5p and miR-497-5p then prompted Endophilin-1 expression that attenuated TJ-related proteins expression and impaired ZO-1 and occludin redistribution. Taken together, these studies suggested that SNHG7 might regulate the function of related miRNAs through ceRNA, thereby affecting the expression of downstream related genes and disease progression.

In summary, our study demonstrated that elevated TARBP2 in Aβ(1–42)-incubated ECs stabilized SNHG7 by prolonging half-life of SNHG7 and SNHG7 regulated NFATC3 expression by acting as ceRNA of miR-17-5p. NFATC3 served as a TF increasing BBB permeability by suppressing TJ-related proteins expression. Considering the early marker roles of BBB leakage, it might suggest a potential mechanism in AD diagnosis and therapy.

## Supplementary information

supplementary figure. S1.tif

supplementary figure. S2

supplementary figure legend

Supplementary table.S1

Original western blots

aj-checklist

## Data Availability

The datasets generated and/or analyzed during the current study are available from the corresponding author on reasonable request.
