# Supplementary figures and images for: TARBP2-stablized SNHG7 regulates blood-brain barrier permeability by acting as a competing endogenous RNA to miR-17-5p/NFATC3 in Aβ-microenvironment
Source: Cell Death Dis. 2022 May 13;13(5):457. doi: 10.1038/s41419-022-04920-8 (PMC9106673; doi:10.1038/s41419-022-04920-8)

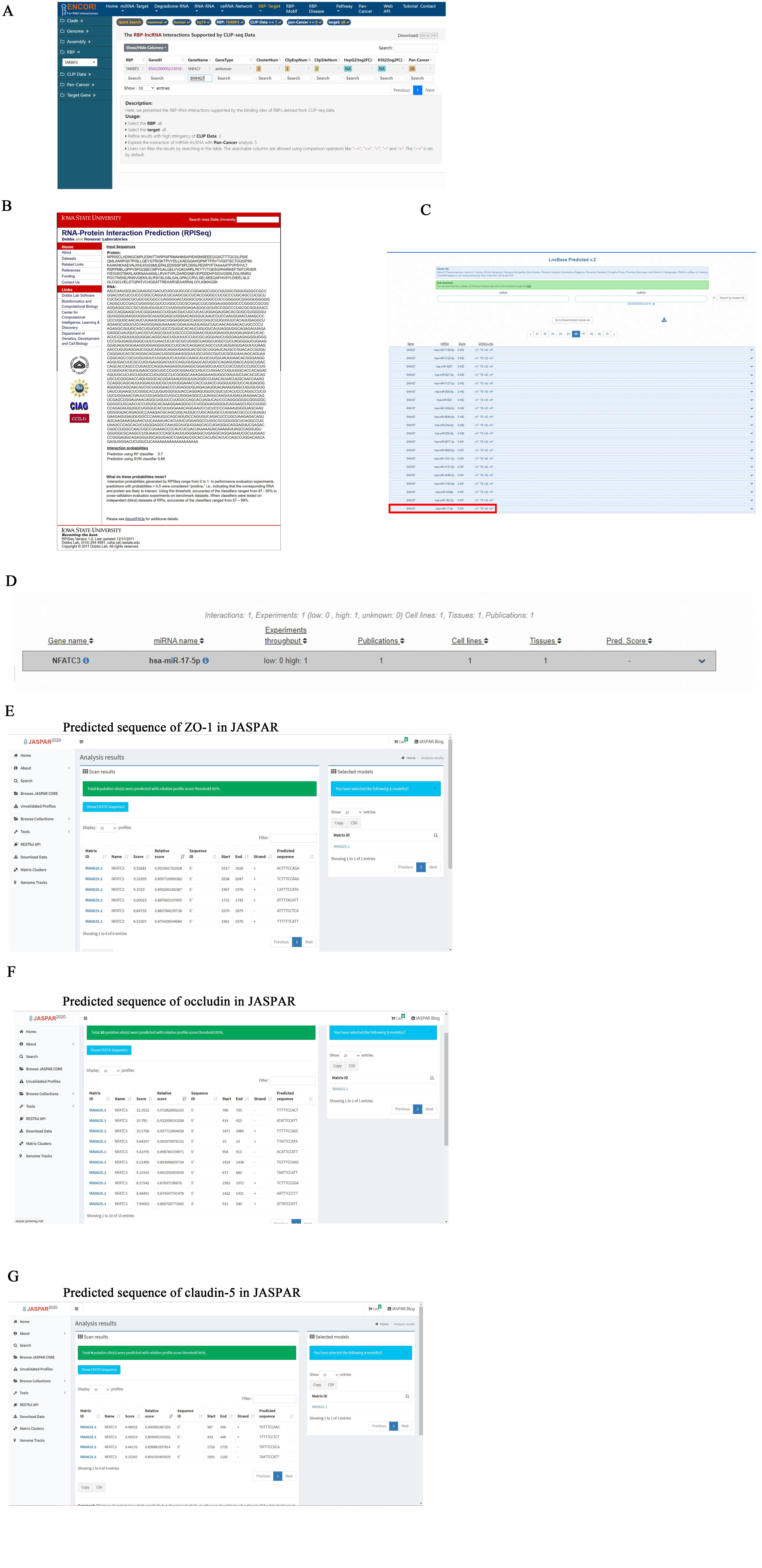

Supplement: Supplementary file 1 — supplementary figure. S1.tif [file 41419_2022_4920_MOESM1_ESM.tif]
