# Supplementary material for: TARBP2-stablized SNHG7 regulates blood-brain barrier permeability by acting as a competing endogenous RNA to miR-17-5p/NFATC3 in Aβ-microenvironment
Source: Cell Death Dis. 2022 May 13;13(5):457. doi: 10.1038/s41419-022-04920-8 (PMC9106673; doi:10.1038/s41419-022-04920-8)
Supplement: Supplementary file 2 — supplementary figure. S2 [file 41419_2022_4920_MOESM2_ESM.pdf]

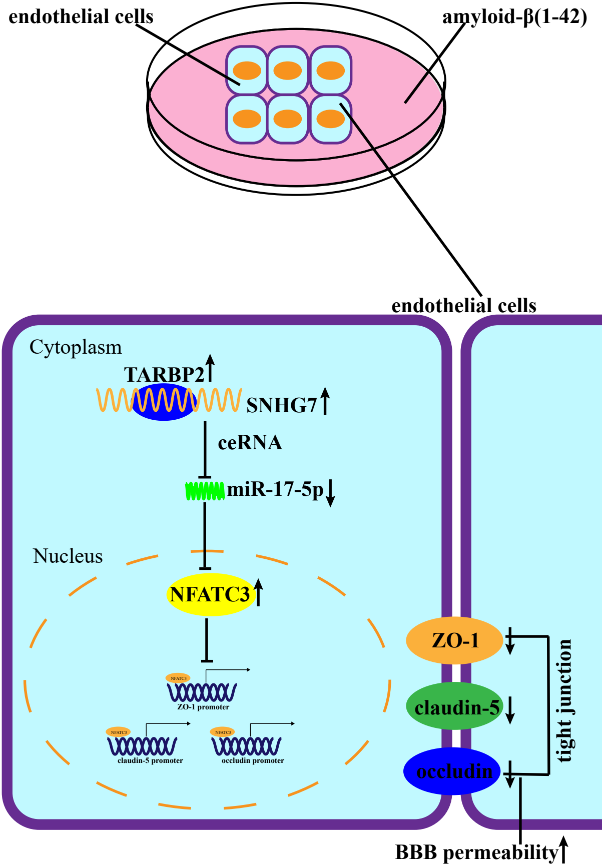
endothelial cells
amyloid-β(1-42)
endothelial cells
Cytoplasm
TARBP2
SNHG7
ceRNA
miR-17-5p
Nucleus
NFATC3
NFATC3
ZO-1 promoter
NFATC3
claudin-5 promoter
NFATC3
occludin promoter
ZO-1
claudin-5
occludin
tight junction
BBB permeability
