# Supplementary material for: TARBP2-stablized SNHG7 regulates blood-brain barrier permeability by acting as a competing endogenous RNA to miR-17-5p/NFATC3 in Aβ-microenvironment
Source: Cell Death Dis. 2022 May 13;13(5):457. doi: 10.1038/s41419-022-04920-8 (PMC9106673; doi:10.1038/s41419-022-04920-8)
Supplement: Supplementary file 3 — supplementary figure legend [file 41419_2022_4920_MOESM3_ESM.docx]

**Figure. S1** **A** **B** Binding relationship between TARBP2 and SNHG7 was predicted by starbase and RPISeq. **C** Binding relationship between SNHG7 and miR-17-5p was predicted by DIANA tool. **D** Binding relationship between miR-17-5p and NFATC3 was predicted by DIANA tool. **E F G** Binding sequences of ZO-1, occludin and claudin-5 to NFATC3 were predicted by JASPAR.

**Figure. S2** Schematic diagram of TARBP2/SNHG7/miR-17-5p/NFATC3 pathway to regulate BBB.
