# Supplementary material for: TARBP2-stablized SNHG7 regulates blood-brain barrier permeability by acting as a competing endogenous RNA to miR-17-5p/NFATC3 in Aβ-microenvironment
Source: Cell Death Dis. 2022 May 13;13(5):457. doi: 10.1038/s41419-022-04920-8 (PMC9106673; doi:10.1038/s41419-022-04920-8)
Supplement: Supplementary file 4 — Supplementary table.S1 [file 41419_2022_4920_MOESM4_ESM.docx]

**A**

Primers and probes used for qRT-PCR

| Primer or Probe | Gene | Sequence (5’->3’) or Assay ID |
| --- | --- | --- |
| Primer | TARBP2 | F: AGGACATTCCGGTTTTTACTGC |
|  |  | R: CTGGGTCACTGTGTACTCCG |
|  | SNHG7 | F: GTGTGTCCCTTGGTGGAGAG |
|  |  | R: TCCCAGATACCAGCGAAGGA |
|  | ZO-1 | F: TGAACGCTCTCATAAGCTTCGTAA |
|  |  | R: ACCGTACCAACCATCATTCATTG |
|  | claudin | F: TCTGCTGGTTCGCCAACAT |
|  |  | R: CGGCACCGTCGGATCA |
|  | occludin | F: TGTGGGATAAGGAACACATTTATGA |
|  |  | R: CAGACACATTTTTAACCCACTCTTCA |
|  | NFATC3 | F: TCGTGTACACATCCCACAGC |
|  |  | R: CAACCATTTCATGACCTCCA |
|  | GAPDH | F:AAATCCCATCACCATCTTCCAG |
|  |  | R:TGATGACCCTTTTGGCTCCC |
|  | mir-17-5p | F:CCAAAGTGCTTACAGTGCAGGTA |

**B**

Sequences of shRNA template

| Gene | Sequence (5’->3’) | |
| --- | --- | --- |
| TARBP2 | Sense | GCTGCCTAGTATAGAGCAAAT |
| SNHG7 | Sense | GGTCAATCCTCCAATGTAACT |
| NFATC3 | Sense | GCTTACCACATCATGGATTAC |

**C**

Wild-type and mutant plasmid sequences

NFATC3

| Wild-type plasmid sequences | Mutant plasmid sequences |
| --- | --- |
| 3’GTTCTCAGTTATGTTTACAGCACTTGGAA5’ | 3’GTTCTCAGTTATGTTTACAGGTACGGGAA5’ |

ZO-1:

| Wild-type plasmid sequences | Mutant plasmid sequences |
| --- | --- |
| 5’TGACTTTCCAGAGA3’ | 5’TGACTTGAATCTGA3’ |
| 5’GTTCTTTCCAAGAC3’ | 5’GTTCTTATTGCAAC3’ |
| 5’TTCATTTCCATATA3’ | 5’TTCATTGTTGCTTA3’ |

occludin:

| Wild-type plasmid sequences | Mutant plasmid sequences |
| --- | --- |
| 5’CCTTTTTCCAGCAA3’ | 5’CCTTTTGAACTTAA3’ |
| 5’TATTATTCCATATG3’ | 5’TATTATGATCGCTG3’ |
| 5’GTATTATCCATTCA3’ | 5’GTATTACAGCGGCA3’ |
| 5’GAAATTTCCCTTGG3’ | 5’GAAATTGTAACGGG3’ |

claudin-5:

| Wild-type plasmid sequences | Mutant plasmid sequences |
| --- | --- |
| 5’TCTTTTTCCTCTCC3’ | 5’TCTTTTGAAGTCCC3’ |
| 5’TCTTTTTCCTCTCC3’ | 5’TCTTTTGAAGAGCC3’ |

**D**

Primers used for ChIP experiments

| Gene | Binding site or control | Sequence (5’->3’) | Product size(bp) | Annealing temperature(°C) |
| --- | --- | --- | --- | --- |
| ZO-1 | PCR1 | TCACTGTGTACTCCGGCAAC | 215 | 59.4 |
|  |  | CCTGGAGGACAGCAGTTCTTT |  |  |
|  | PCR2 | TGCAGCAAAGAAATAAAAAAATGGTAA | 209 | 60.1 |
|  |  | ACCTCATTTCCTCCGGG |  |  |
|  | PCR3 | AATTCTCAATGTTTGAATGTTTTAAACTGA | 240 | 59.4 |
|  |  | GGAGAATGAGGGGGGAGA |  |  |
|  | PCR4 | TCCAGAGACTAGATATGCAGTCA | 229 | 60.0 |
|  |  | AGTGTAACTCTTATATACCACCTTTTCT |  |  |
|  | PCR5 | TTTACAAACACAAAAAATACTTTAATTCTCAATGT | 222 | 57.8 |
|  |  | GAATCACTTGAGCCCCGG |  |  |
| occludin | PCR1 | GTCCCTCTACTCGCGAGATTC | 226 | 57.6 |
|  |  | ATGCAGACACTCCCTGCTTC |  |  |
|  | PCR2 | CCTCATTTTAACCCCTCTAAGTAATTGT | 235 | 59.4 |
|  |  | CGAACGAGGTCCAGAGGG |  |  |
|  | PCR3 | TGAATCCTATTATTCCATATGGATGAACTC | 240 | 60.3 |
|  |  | CATTTTAAGATAACTTATGCATATCATGTTATTTTTC |  |  |
|  | PCR4 | TATTATTCAGTTATAAAAAGGAATGAAGTACCAAT | 225 | 60 |
|  |  | CCAAAAGAAACCCCATGCC |  |  |
|  | PCR5 | CCAAGAACTATAATTGCCACATCC | 75 | 59.1 |
|  |  | CACTACTTAAAAAGCAAAATAAACTTGGT |  |  |
| claudin-5 | PCR1 | ATTGGAACGATACAGAGAAGATT | 227 | 59.4 |
|  |  | GGAACGCTTCACGAATTTG |  |  |
|  | PCR2 | CTCTGCACACAATGTGTCTTTTTCC | 235 | 58.5 |
|  |  | ATGGAGAAACCCCGTCTCTACT |  |  |
|  | PCR3 | TTTGGGTGCTTGGGCGG | 240 | 58.8 |
|  |  | GAATCAAGAAGAAGGAGATTTCTTGTTGGAAAC |  |  |
