# Supplementary material for: TARBP2-stablized SNHG7 regulates blood-brain barrier permeability by acting as a competing endogenous RNA to miR-17-5p/NFATC3 in Aβ-microenvironment
Source: Cell Death Dis. 2022 May 13;13(5):457. doi: 10.1038/s41419-022-04920-8 (PMC9106673; doi:10.1038/s41419-022-04920-8)
Supplement: Supplementary file 5 — Original western blots [file 41419_2022_4920_MOESM5_ESM.pdf]

Relative expression of TARBP2

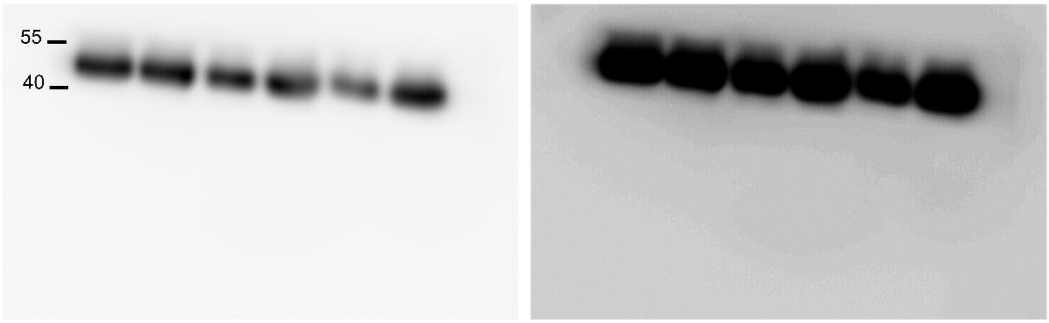

GAPDH of relative expression of TARBP2

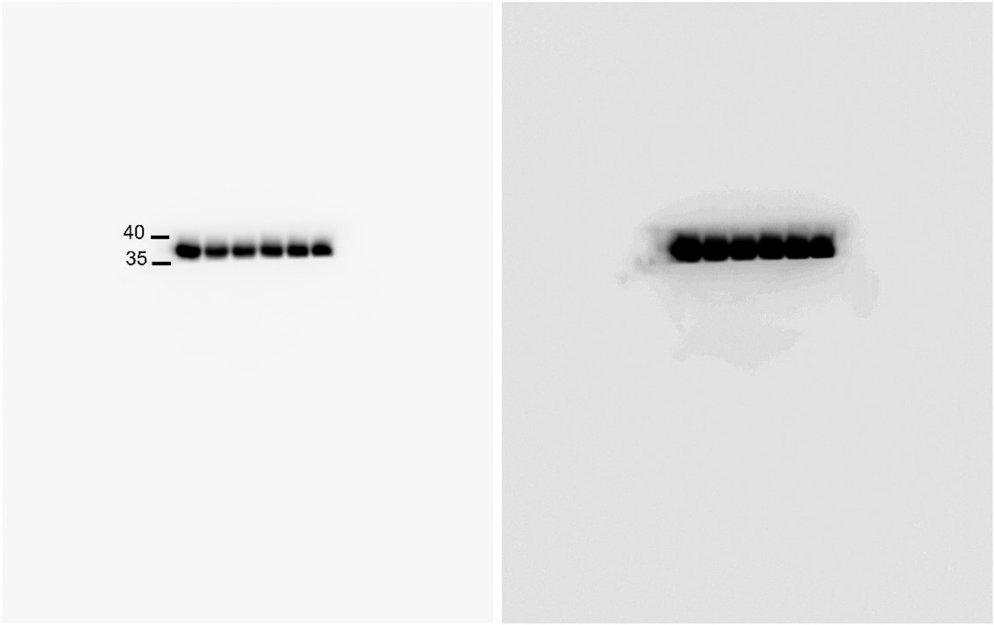

ZO-1 of overexpression/knockdown of TARBP2

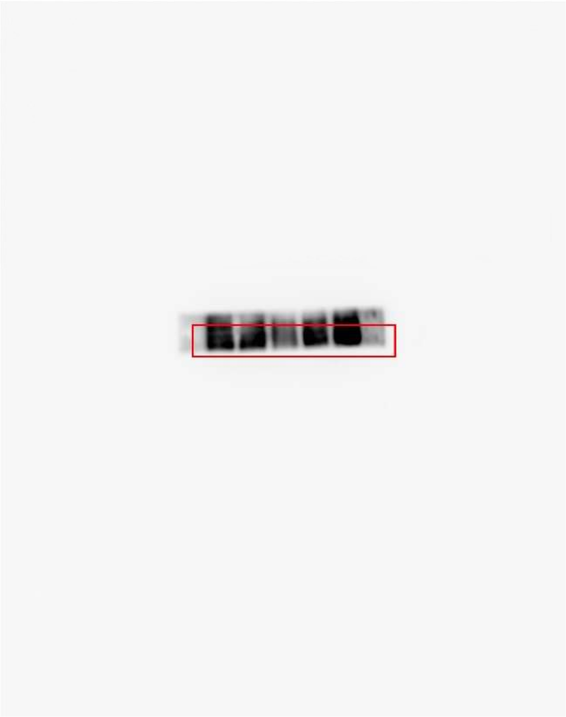

occludin of overexpression/knockdown of TARBP2

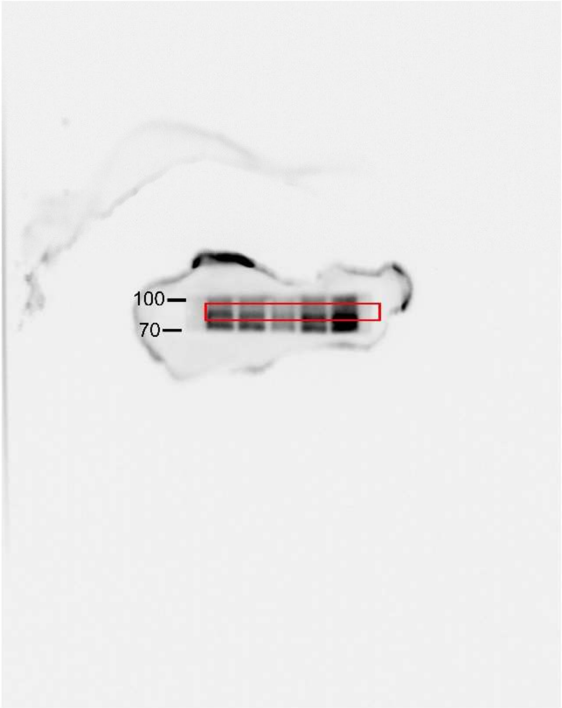

claudin-5 of overexpression/knockdown of TARBP2

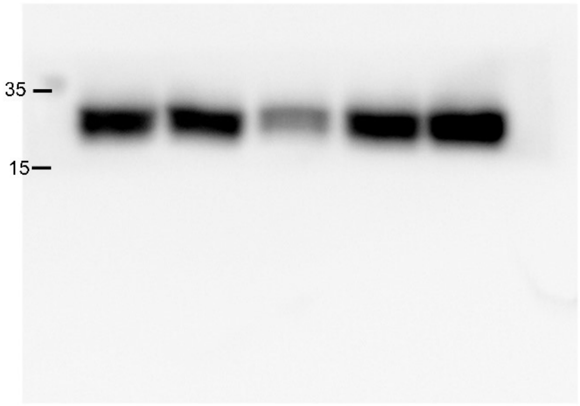

GAPDH of TJ-related proteins overexpression/knockdown of TARBP2

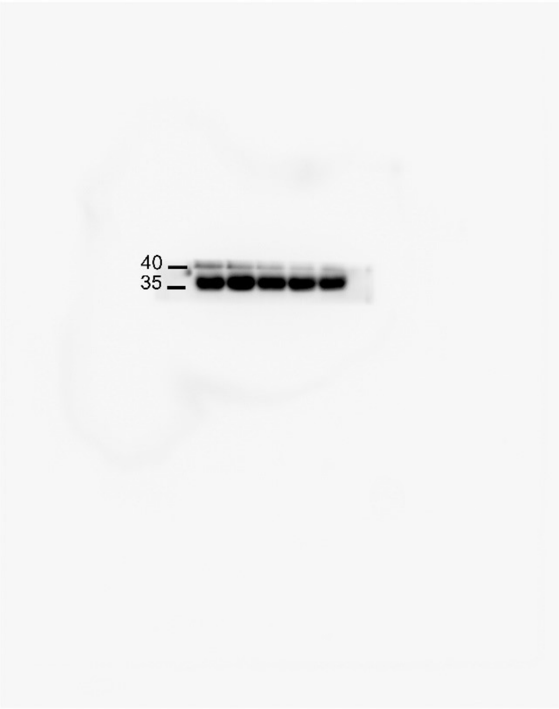

NFATC3 of overexpression/knockdown of TARBP2

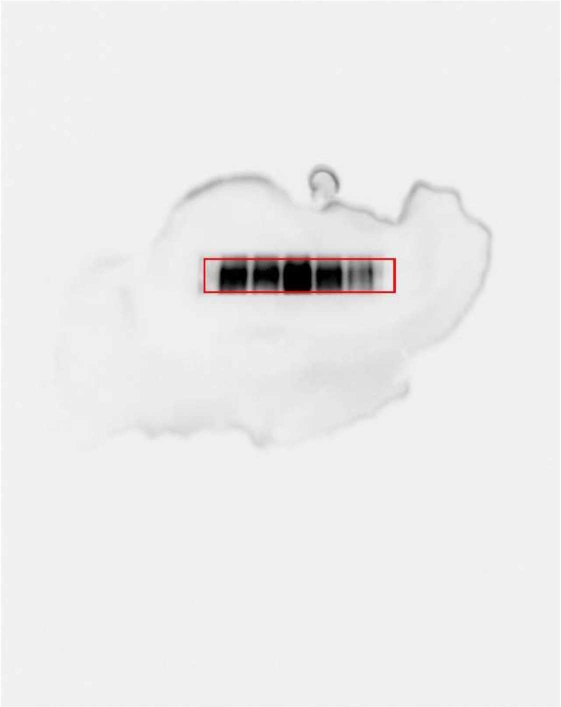

GAPDH of NFATC3 of overexpression/knockdown of TARBP2

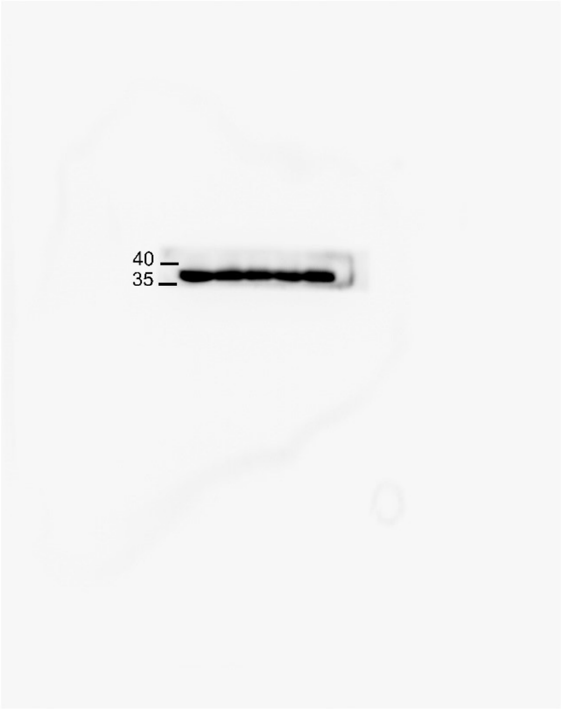

ZO-1 of overexpression/knockdown of SNHG7

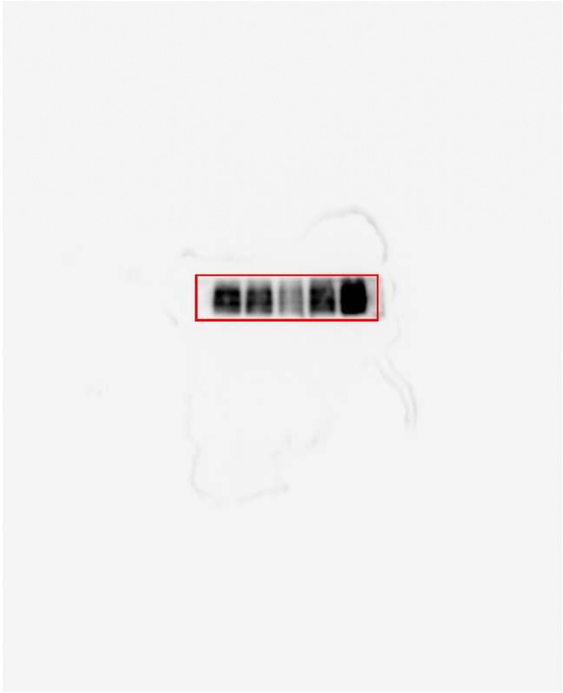

occludin of overexpression/knockdown of SNHG7

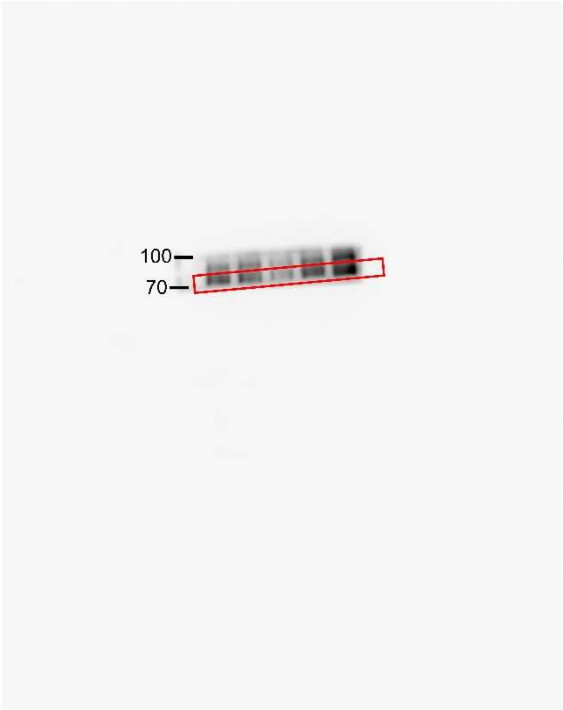

claudin-5 of overexpression/knockdown of SNHG7

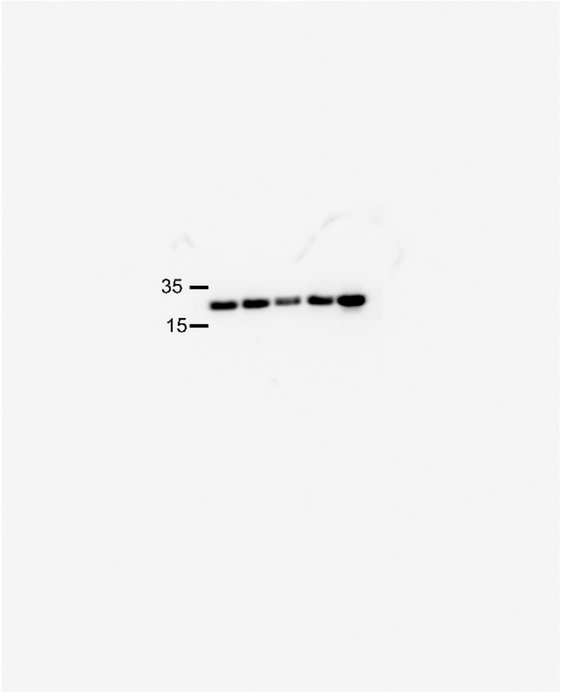

GAPDH of TJ-related proteins overexpression/knockdown of SNHG7

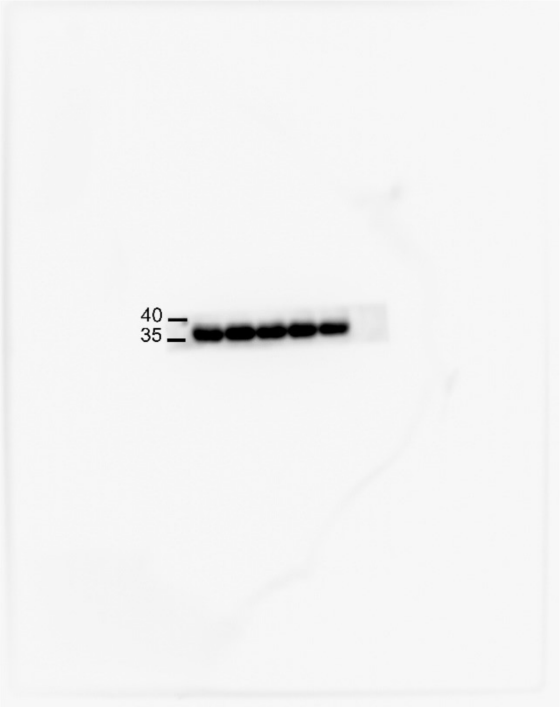

NFATC3 of overexpression/knockdown of SNHG7

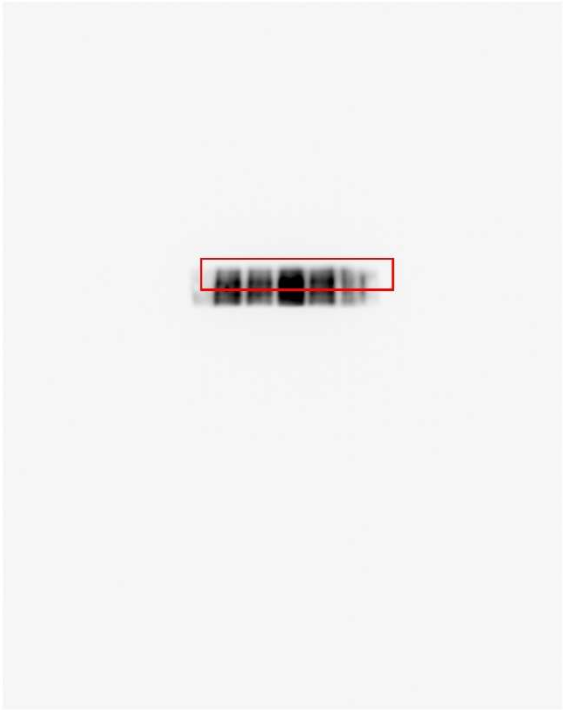

GAPDH of NFATC3 of overexpression/knockdown of SNHG7

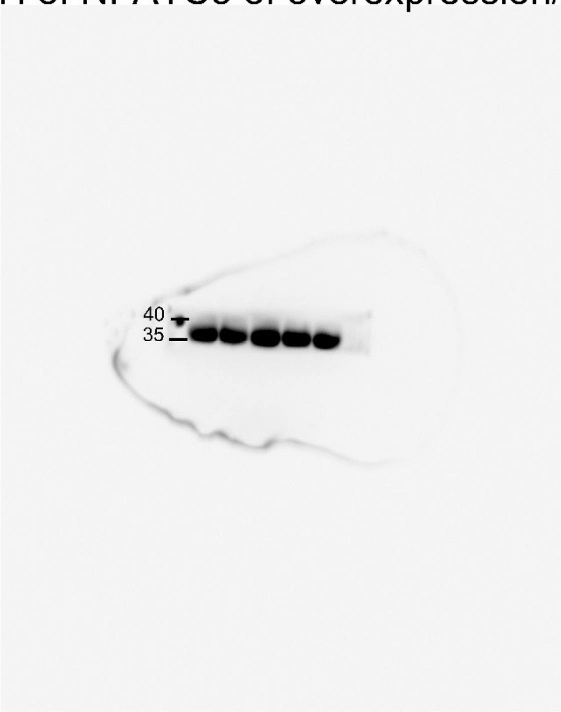

TJ-related proteins of TARBP2(+)+SNHG7(-)

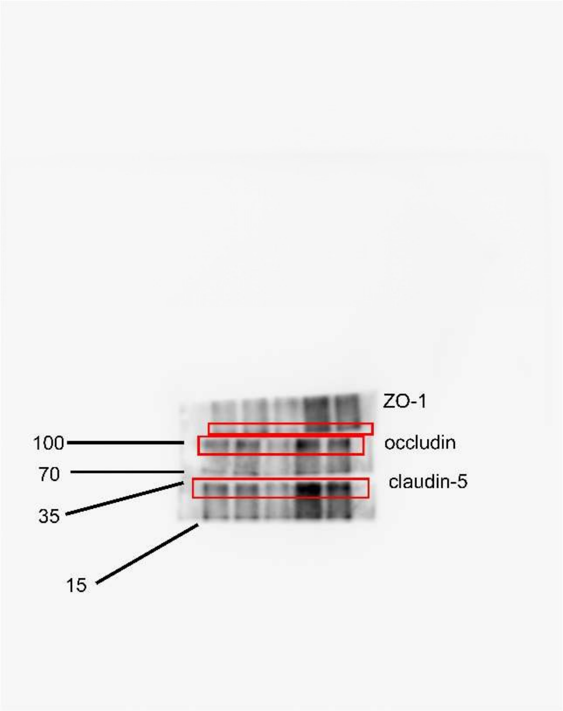

GAPDH of TJ-related proteins of TARBP2(+)+SNHG7(-)

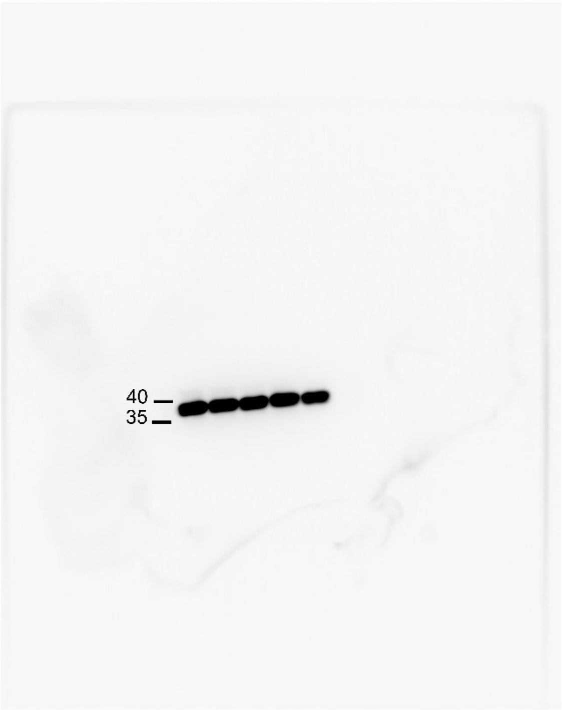

RNA pull down of TARBP2

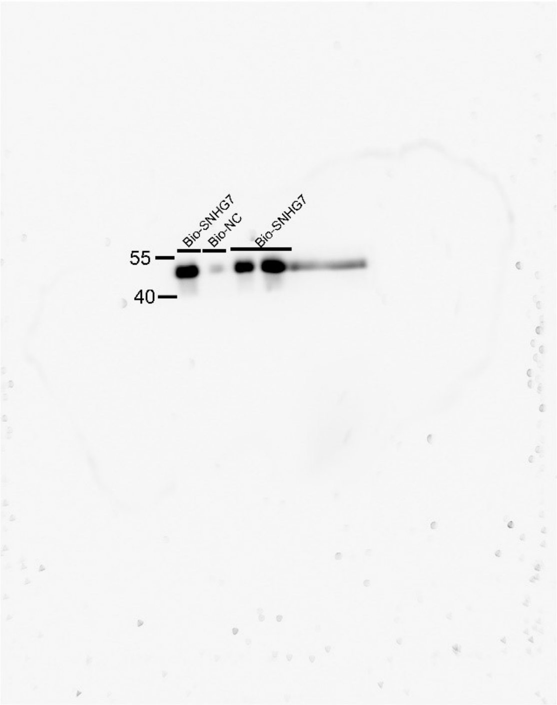

GAPDH of RNA pull down of TARBP2

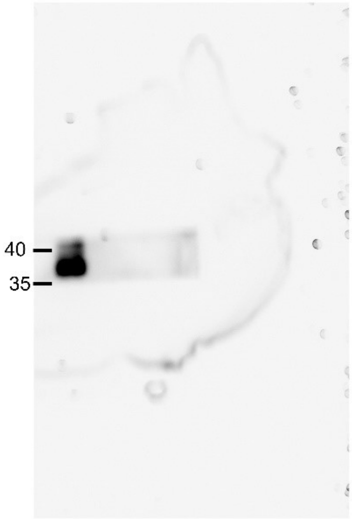

ZO-1 of overexpression/knockdown of miR-17-5p

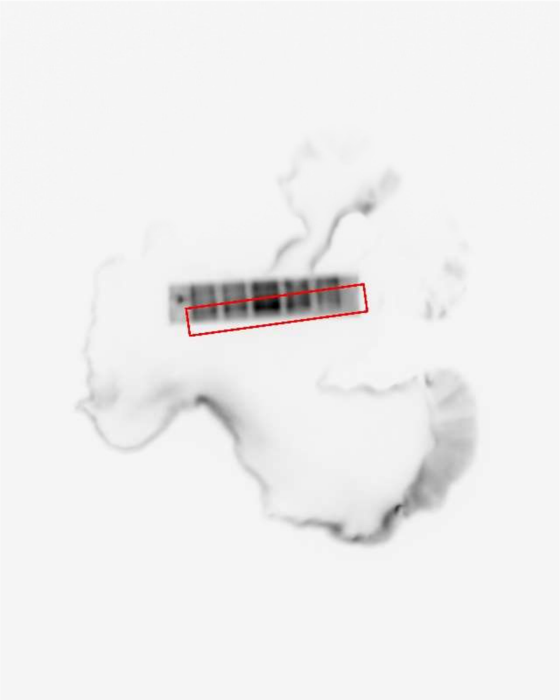

occludin of overexpression/knockdown of miR-17-5p

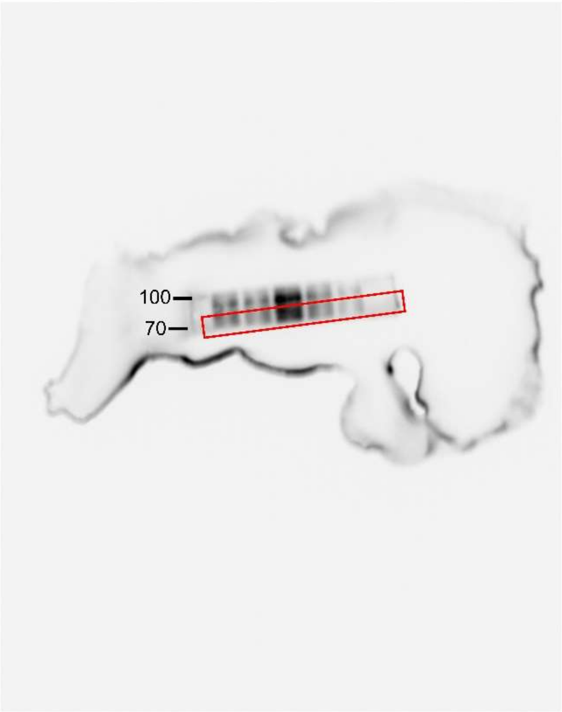

claudin-5 of overexpression/knockdown of miR-17-5p

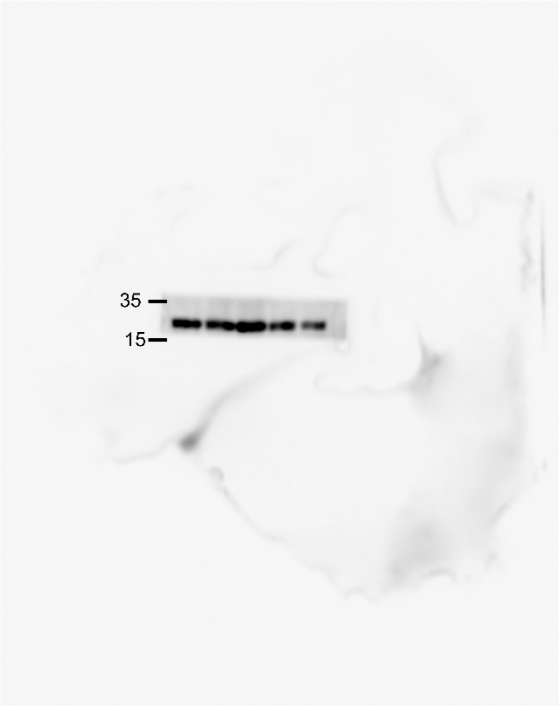

GAPDH of TJ-related proteins overexpression/knockdown of miR-17-5p

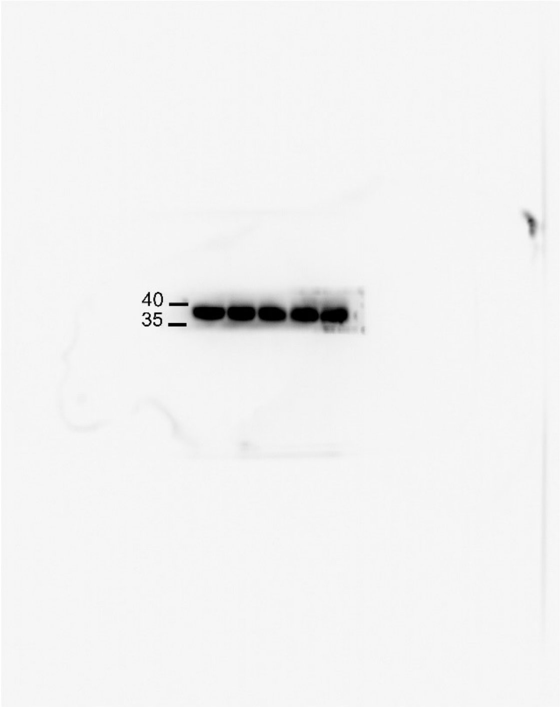

NFATC3 of overexpression/knockdown of miR-17-5p

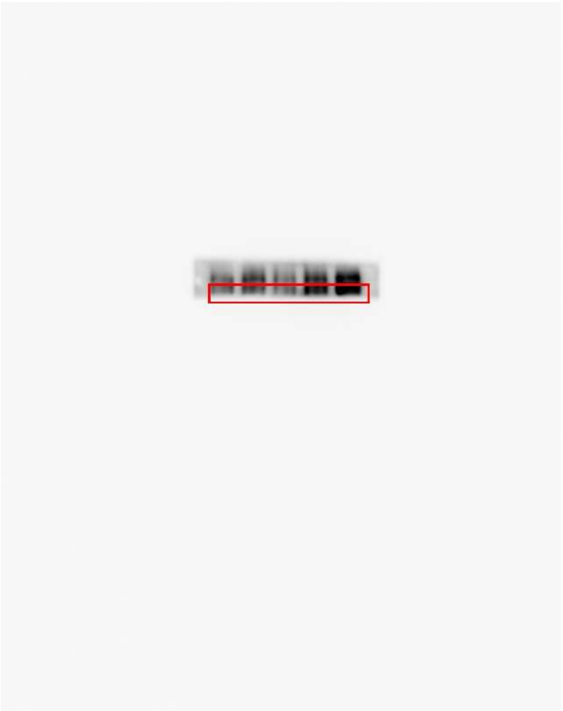

GAPDH of NFATC3 of overexpression/knockdown of miR-17-5p

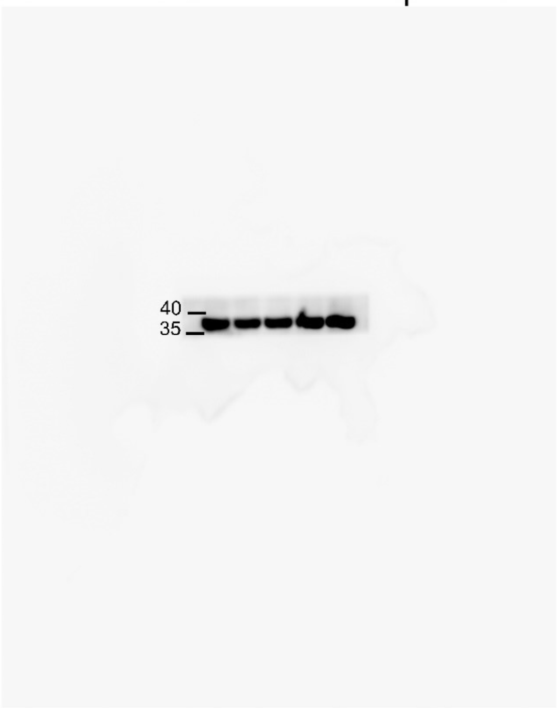

Relative expression of NFATC3

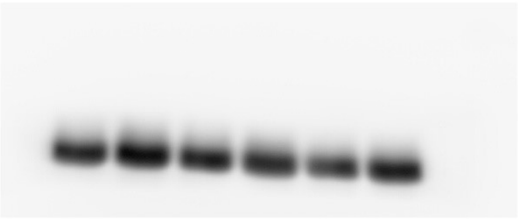

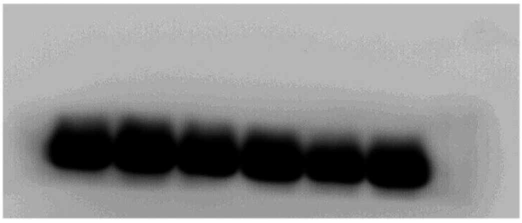

GAPDH of relative expression of NFATC3

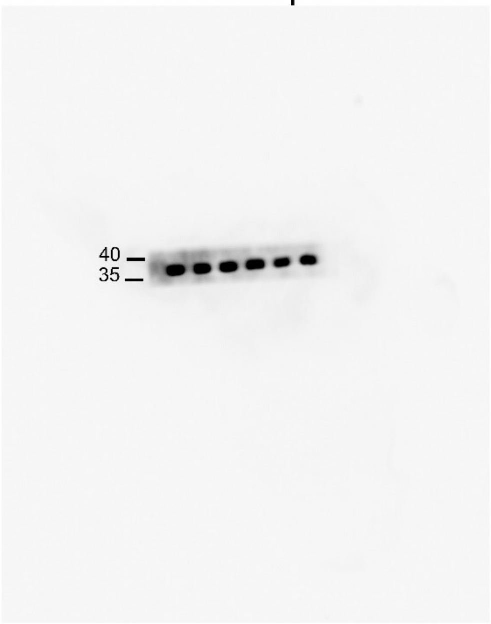

ZO-1 of overexpression/knockdown of NFATC3

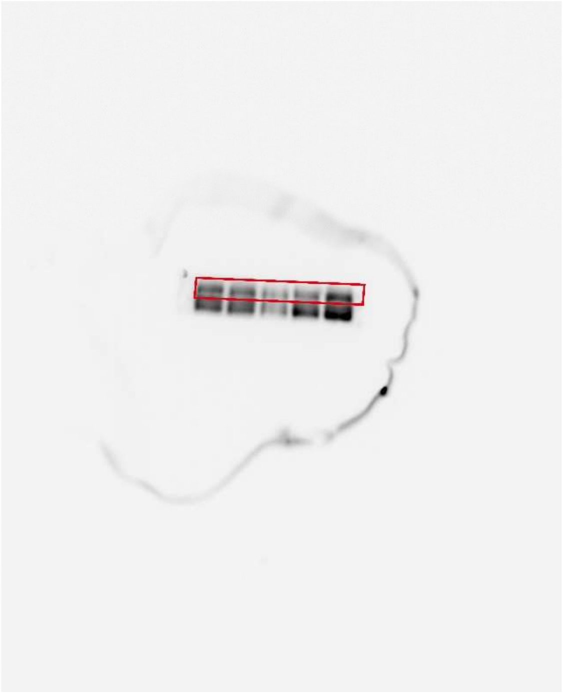

occludin of overexpression/knockdown of NFATC3

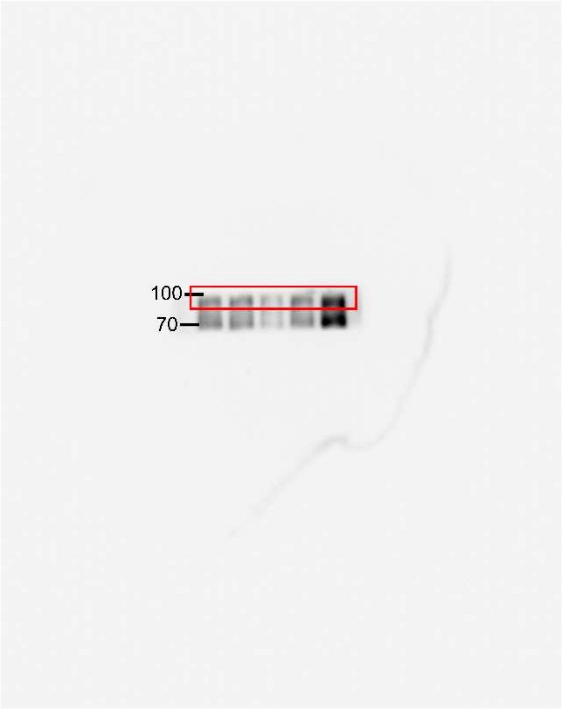

claudin-5 of overexpression/knockdown of NFATC3

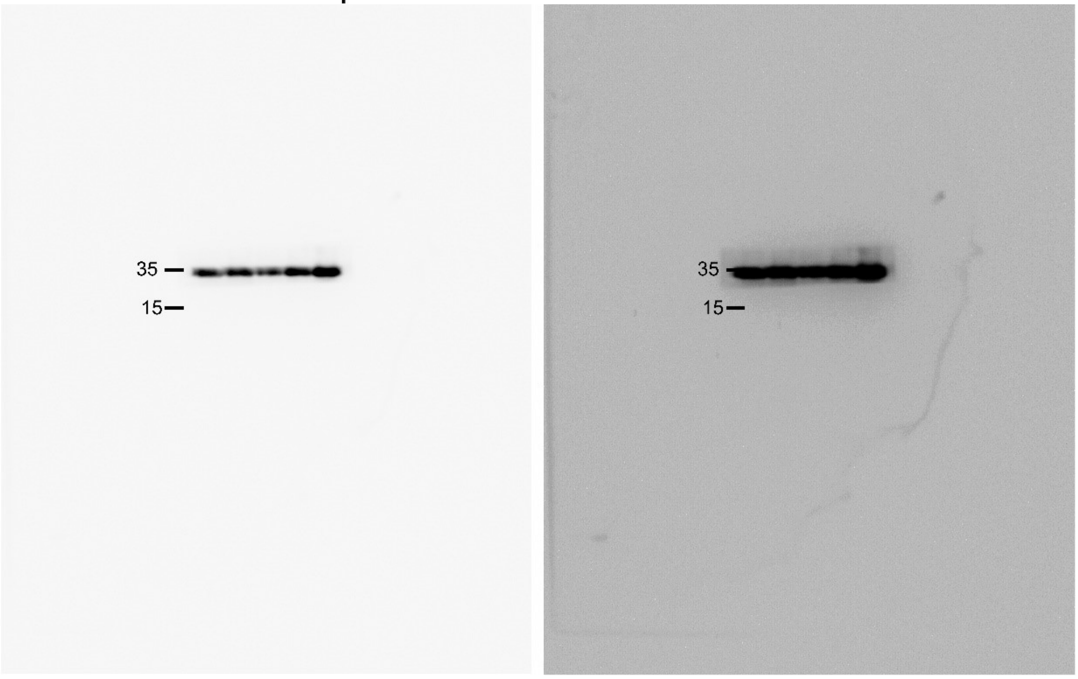

GAPDH of TJ-related proteins overexpression/knockdown of NFATC3

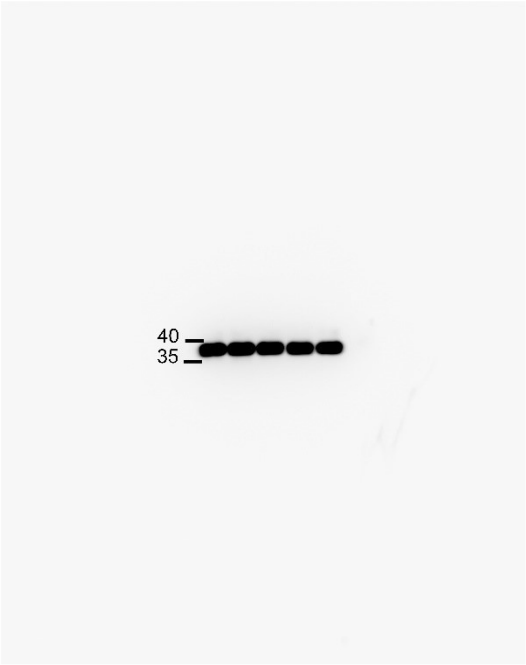

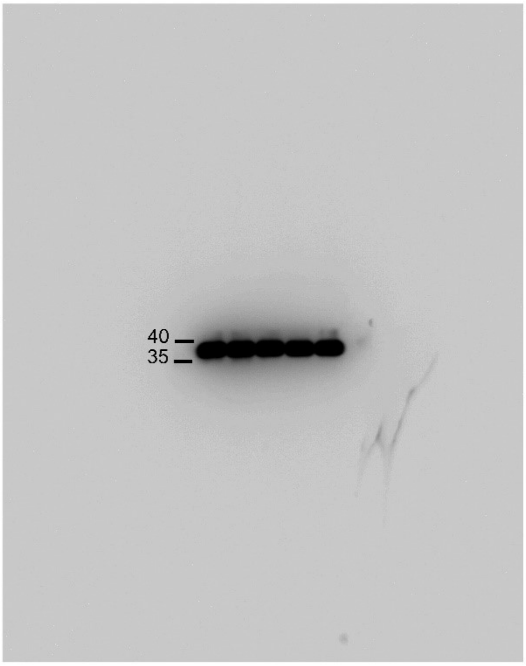

NFATC3 of miR-17-5p+SNHG7

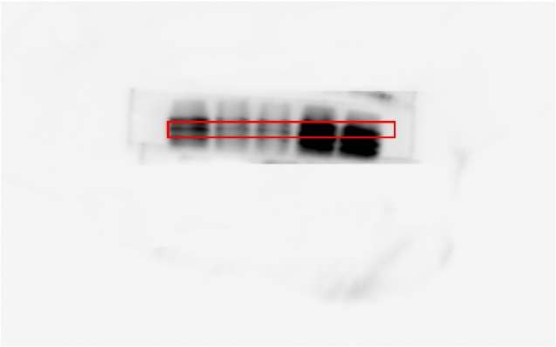

GAPDH of NFATC3 of miR-17-5p+SNHG7

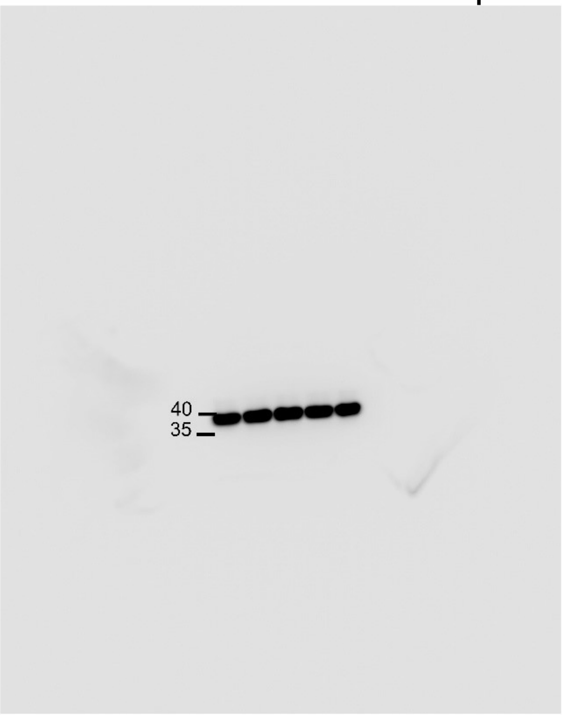

The original western blot pictures are shown. Because of the characteristics of monoclonal antibodies we used, the background of the ECL luminescence of some proteins was relatively clean. So, color gradation in Relative expression of TARBP2, GAPDH of Relative expression of TARBP2, Relative expression of NFATC3, claudin-5 of overexpression/knockdown of NFATC3 and claudin-5 of overexpression/knockdown of NFATC3 was adjusted to show peripheral ECL Substrate imprints more clearly and pictures were presented at right sides. In other words, we provided two different backgrounds of the same image to prove that we had not done any cropping to the original image.

All uncropped original western blot pictures were imaged by MicroChemi 4.2 instrument (DNR, ISRAEL) with ECL chemiluminescence kit (Beyotime, Shanghai).
